# H3K9me3-binding proteins are dispensable for SETDB1/H3K9me3-dependent retroviral silencing

**DOI:** 10.1186/1756-8935-4-12

**Published:** 2011-07-20

**Authors:** Irina A Maksakova, Preeti Goyal, Jörn Bullwinkel, Jeremy P Brown, Misha Bilenky, Dixie L Mager, Prim B Singh, Matthew C Lorincz

**Affiliations:** 1 Department of Medical Genetics, Life Sciences Institute, University of British Columbia, 2350 Health Sciences Mall, Vancouver, BC, Canada, V6T 1Z3; 2 Terry Fox Laboratory, BC Cancer Agency, 675 West 10th Avenue, Vancouver, BC, Canada, V5Z 1L3; 3 Division of Immunoepigenetics, Department of Immunology and Cell Biology, Research Center Borstel, Parkallee 22, D-23845 Borstel, Germany; 4 Canada's Michael Smith Genome Sciences Centre, BC Cancer Agency, 675 West 10th Avenue, Vancouver, BC, V5Z 1L3, Canada

**Keywords:** endogenous retrovirus, ERV, heterochromatin protein 1, HP1, *Cbx1*, *Cbx3*, *Cbx5*, H3K9me3, retroviral repression, transcriptional silencing, mouse embryonic stem cells

## Abstract

**Background:**

Endogenous retroviruses (ERVs) are parasitic sequences whose derepression is associated with cancer and genomic instability. Many ERV families are silenced in mouse embryonic stem cells (mESCs) via SETDB1-deposited trimethylated lysine 9 of histone 3 (H3K9me3), but the mechanism of H3K9me3-dependent repression remains unknown. Multiple proteins, including members of the heterochromatin protein 1 (HP1) family, bind H3K9me2/3 and are involved in transcriptional silencing in model organisms. In this work, we address the role of such H3K9me2/3 "readers" in the silencing of ERVs in mESCs.

**Results:**

We demonstrate that despite the reported function of HP1 proteins in H3K9me-dependent gene repression and the critical role of H3K9me3 in transcriptional silencing of class I and class II ERVs, the depletion of HP1α, HP1β and HP1γ, alone or in combination, is not sufficient for derepression of these elements in mESCs. While loss of HP1α or HP1β leads to modest defects in DNA methylation of ERVs or spreading of H4K20me3 into flanking genomic sequence, respectively, neither protein affects H3K9me3 or H4K20me3 in ERV bodies. Furthermore, using novel ERV reporter constructs targeted to a specific genomic site, we demonstrate that, relative to *Setdb1*, knockdown of the remaining known H3K9me3 readers expressed in mESCs, including *Cdyl*, *Cdyl2*, *Cbx2*, *Cbx7*, *Mpp8*, *Uhrf1 and Jarid1a-c*, leads to only modest proviral reactivation.

**Conclusion:**

Taken together, these results reveal that each of the known H3K9me3-binding proteins is dispensable for SETDB1-mediated ERV silencing. We speculate that H3K9me3 might maintain ERVs in a silent state in mESCs by directly inhibiting deposition of active covalent histone marks.

## Background

Endogenous retroviral sequences (ERVs) are relics of ancient retroviral integration into the germline. These parasitic elements are abundant in mammals, occupying approximately 8% of the mouse genome and 10% of the human genome [1,2]. ERVs are subdivided into three diverse classes based on the similarity of their reverse transcriptase genes or their relationship to different genera of exogenous retroviruses. In the mouse, class I ERVs, similar to gammaretroviruses, include active families such as murine leukaemia viruses (MLVs) and murine retroviruses that use tRNA^Gln ^(GLN). Class II ERVs are similar to alpha- and betaretroviruses and include *Mus musculus *ERV using tRNA^Lys ^type 10C (MMERVK10C), the highly retrotranspositionally active intracisternal A-type particles (IAPEz) and early transposon/*Mus musculus *type D retrovirus (ETn/MusD) families. Class III ERVs, the oldest and most abundant ERVs, are most similar to spumaviruses and are represented by mouse endogenous retrovirus type L (MERV-L) and mouse apparent LTR retrotransposons (MaLR) [3,4]. Numerous regulatory motifs in the ERV long terminal repeats (LTRs) can initiate high levels of transcription in tissues and cell lines [5], and there is extensive evidence of aberrant ERV-driven gene expression in cancers [6-11] and tissues of aging mice [12,13]. In an effort to counteract the potentially detrimental effects of ERVs, eukaryotic genomes have evolved multiple lines of defence against active exogenous and endogenous retroviruses [14], including DNA methylation and repressive histone modifications.

DNA methylation was the first epigenetic mark recognized to contribute to ERV silencing, with dramatic upregulation of ERVs observed in DNA methylation-deficient somatic cells [15,16]. However, genome-wide chromatin immunoprecipitation (ChIP) followed by ChIP sequencing (ChIP-seq) [17-19] or ChIP followed by quantitative PCR (qPCR) [20] revealed that in mouse embryonic stem cells (mESCs), class I and class II ERVs are enriched for the repressive histone H3 lysine 9 trimethylation (H3K9me3) deposited by lysine methyltransferase (KMTase) SETDB1/ESET/KMT1E [20]. SETDB1 is in turn thought to be recruited to ERVs via the obligatory corepressor KRAB-associated protein 1 (KAP-1) [21], presumably through sequence-specific KAP-1-binding zinc finger proteins such as ZFP809 in the case of MLVs [22]. Moreover, we and others have recently shown that in mESCs, H3K9me3 and SETDB1 play a greater role than DNA methylation in the silencing of class I and class II ERVs [20,23]. IAP and ETn/MusD retrotransposons, the two most active class II mouse ERV families and the source of numerous recent germline mutations [24], are among the families with the highest H3K9me3 enrichment levels. Intriguingly, these families are dramatically upregulated in SETDB1 knockout (SETDB1 KO) mESCs [19,20], confirming that they have a high potential for activation in the absence of H3K9me3. In contrast, the class III MERV-L and MaLR families, which are devoid of the H3K9me3 mark in mESCs, are repressed by the histone lysine-specific demethylase 1 (LSD1/KDM1A) [25], revealing that different ERV classes are regulated by distinct epigenetic modifications in these pluripotent cells.

Acetylation of lysine residues on the N-terminal tails of histones, including H3K9, directly influences the state of chromatin compaction by reducing the affinity of histones for DNA [26,27]. In contrast, methylation *per se *of such lysine residues is less likely to directly affect chromatin structure, as this modification does not alter their charge. Rather, the prevailing view is that specific proteins, the so-called "readers," bind to methylated lysines and coordinate the biological outcome associated with such covalent histone marks. H3K9me3, for example, which is essential for the establishment and maintenance of the silent chromatin state [28-31], is bound by three isoforms of heterochromatin protein 1 (HP1) in the mouse genome: HP1α (encoded by *Cbx5*), HP1β (encoded by *Cbx1*) and HP1γ (encoded by *Cbx3*) [32]. HP1 is a highly conserved family; its members are frequently present in several copies in eukaryotic genomes and play both structural and gene regulatory roles [33-35]. The chromodomain of HP1 is responsible for binding H3K9me2/3 [36,37], and a chromoshadow domain is required for HP1 homo- and heterodimerization and the recruitment of other proteins [38,39].

Although their exact function in transcriptional regulation and cross-talk with histone and DNA methylation varies between species, the ability of HP1s to modulate gene expression via H3K9me2/3 binding has been reported in multiple systems [33,40-42]. In fission yeast, for example, two HP1 homologues, Swi6 and Chp2, are both required for assembly of repressive chromatin [43]. In mammalian cells, targeting of HP1α, HP1β and HP1γ to heterologous loci is sufficient to induce recruitment of SETDB1 and deposition of H3K9me3 [44], and HP1 has been implicated in SUV39H1-mediated silencing of euchromatic genes [45].

A role for HP1 proteins in silencing of repetitive and/or transposable elements has been well documented in several model organisms. In *Drosophila*, two families of transposons are derepressed in larvae with mutant HP1a and, to a lesser extent, mutant HP1c [46]. HP1d/Rhino is required for transposon silencing in the female germline of *Drosophila*, but this silencing seems to stem from Rhino's role in Piwi-interacting RNA (piRNA) production rather than establishment of repressive chromatin [47]. At transposable elements in *Neurospora*, DNA methylation is dependent on methylated H3K9 bound by HP1 [48,49]. In *Arabidopsis*, however, H3K9me3-directed DNA methylation applies only to CpNpG methylation, not to CpG methylation, of transposons [50,51]. HP1γ is a negative regulator of HIV in human cell lines [52] and of non-LTR LINE1 retrotransposons in male mouse germ cells [53]. On the contrary, HP1γ has also been implicated in activating gene expression through its association with elongating RNA polymerase II [54,55]. The latter example notwithstanding, HP1 proteins are excellent candidates for the role of downstream effectors of H3K9me3-dependent silencing affecting ERVs in mESCs. Indeed, an intact HP1-binding domain of KAP-1 is essential for complete restriction of MLV in mouse embryonic carcinoma cells [56]. Furthermore, direct interaction of HP1 and KAP-1, as well as binding of HP1 to H3K9me3, is necessary for the full extent of silencing mediated by these factors [57-61]. Moreover, we have recently demonstrated by ChIP-qPCR that HP1α, HP1β and HP1γ are enriched on IAPEz, MusD and MLV ERV sequences in mESCs, albeit at modest levels, and that this binding is partially dependent on SETDB1-deposited H3K9me3 [20]. On the basis of these observations, we hypothesized that HP1s might play a role in H3K9me3-mediated ERV silencing in mESCs and possibly in early embryos.

In addition to their reported roles in transcriptional silencing, HP1 proteins are required for heterochromatin spreading in specific genomic contexts in *Drosophila *[62,63], yeast [64] and mammals [42,57]. The presence of both chromodomains and chromoshadow domains suggests that HP1 proteins may bind H3K9me3 and recruit additional proteins, such as SUV39H1/2 or SETDB1-bound KAP-1 [61,65,66], to facilitate the spreading of the repressive H3K9me3 mark [67,68]. Intriguingly, repetitive elements may act as foci of *de novo *heterochromatin formation and spreading, as H3K9me3 is enriched at sequences flanking ERVs [18,19]. Conversely, in *Neurospora*, HP1 is a component of a histone demethylase-containing complex that prevents spreading of heterochromatin [69].

In addition to HP1s, many other mouse chromodomain proteins [70] are reported to bind H3K9me3 *in vitro*, including CDYL, CDYL2, CBX2, CBX4, CBX7 and M-phase phosphoprotein 8 (MPP8) [71-78]. Furthermore, nonchromodomain proteins with affinity for H3K9me3 have also been identified [79]. Although MPP8 and CBX7 have been shown to negatively influence transcription of specific genes [71,80], the functional and biological significance of the interaction of most of these H3K9me3 readers with H3K9me3 remains poorly understood.

To determine what role, if any, H3K9me3 readers play in silencing of ERVs and spreading of repressive chromatin from these repetitive elements, we first generated *Cbx1 *(HP1β) knock-out (KO) and *Cbx5 *(HP1α) KO mESCs [40,81]. Surprisingly, we observed no upregulation of ERVs in *Cbx5*^-/- ^mESCs and only modest upregulation of several ERV families in *Cbx1*^-/- ^mESCs compared to that seen in *Setdb1 *KO mESCs. We found that both HP1α and HP1β are dispensable for DNA methylation of the ETnII/MusD family of ERVs, although HP1α has a modest influence on DNA methylation of IAP elements. Furthermore, we demonstrate that while deposition of H4K20me3 at major satellite repeats is dependent in part on HP1α, as reported previously [82], HP1α and HP1β are dispensable for deposition of H4K20me3 at ERVs and play only a modest role in spreading of H4K20me3 into sequences flanking these elements. Finally, employing RNAi and newly derived mESC lines harbouring silenced IAP, MusD and exogenous MLV-based reporters, we show that depletion of all of the HP1 proteins, alone or in combination, or each of the remaining known H3K9me3-binding proteins, has only a modest effect on ERV derepression, indicating that at classes I and II ERVs, H3K9me3 inhibits transcription independently of HP1 and other known H3K9me3 readers.

## Results

### Catalytic activity of SETDB1 is largely required for ERV silencing

**Figure 1:**
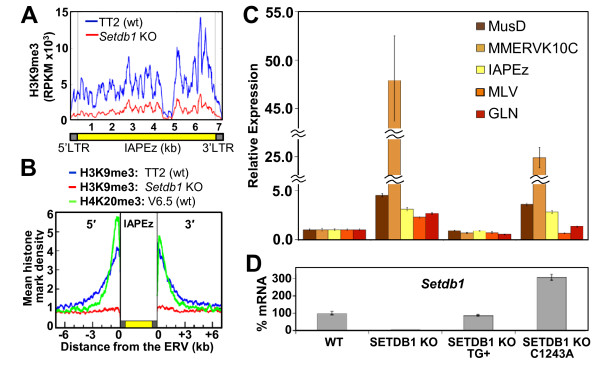
**Catalytically active SETDB1 is required for endogenous retrovirus silencing**. **(A) **Profiling of trimethylated lysine 9 of histone 3 (H3K9me3) along the length of IAPEz endogenous retroviruses (ERVs) in the TT2 wild type (TT2 wt) and *Setdb1 *knockout (*Setdb1 *KO) mouse embryonic stem cells (mESCs) (see Figure S3 in Additional file 1 for profiles of murine leukaemia virus (MLV), MusD, MMERVK10C and GLN ERVs). The profile was generated by aligning chromatin immunoprecipitation assay sequencing (ChIP-seq) reads from TT2 wt and *Setdb1 *KO mESCs [19] to the consensus sequence of IAPEz. H3K9me3 enrichment levels are presented as reads per kilobase per million mapped reads values (RPKM). **(B) **Profiling of H3K9me3 and H4K20me3 in the genomic regions flanking 599 IAPEz elements in TT2 wt and *Setdb1 *KO mESCs (see Figure S4 in Additional file 1 for MusD and MLV profiles). H3K9me3 ChIP-seq reads from TT2 wt (C57BL/6 ± CBA) and *Setdb1 *KO mESCs [19] were used, along with H4K20me3 ChIP-seq from the wt V6.5 mESCs (129SvJae ± C57BL/6) [18]. Reads were aligned to the mouse genome (*mm9*), and the density of reads mapping to the 7-kb regions flanking intact IAPEz ERV families was plotted for H3K9me3 in TT2 wt and *Setdb1 *KO mESCs and for H4K20me3 in V6.5 wt mESCs. Vertical lines indicate the 5' and 3' boundaries of the ERV. The average mappability for 50-bp reads was confirmed to be, on average, uniform in the assayed 7 kb region (data not shown), ruling out the possibility of mapping bias. **(C) ***Setdb1 *deletion was induced with **4-hydroxytamoxifen **(4-OHT) in mESCs containing no transgene (KO), a wt transgene (KO TG+) or a transgene with a mutation rendering SETDB1 catalytically inactive (KO C1243A) [20]. Expression is normalized to *β-actin *relative to wt. Data are presented as means ± standard deviations (SD) for three technical replicates. **(D) **To establish the expression levels of *Setdb1 *in the KO and transgenic lines, quantitative RT-PCR (qRT-PCR) was performed with *Setdb1*-specific primers, and expression was normalized to *β-actin *relative to wt. Data are presented as means ± SD for three technical replicates.

We recently showed by ChIP-qPCR [20] and ChIP-seq [19] analyses that numerous class I and class II ERV families are marked by H3K9me3. Furthermore, we demonstrated the critical role of SETDB1, the KMTase that deposits this mark, in transcriptional repression of these ERVs. Mapping all H3K9me3 ChIP-seq reads along the span of the consensus sequences of class I and class II ERVs, including IAPEz, MusD, MMERVK10C, MLV and GLN, confirms a high but nonuniform level of H3K9me3 along these elements in wt mESCs and a significantly lower level of H3K9me3 in *Setdb1 *KO mESCs (Figure 1A and Figure S3 in Additional file 1). Consistent with these data and those published in a previous report [18], analysis of the uniquely mapped ChIP-seq reads reveals a high level of H3K9me3 in the regions flanking IAPEz, MusD and MLV ERVs (Figure 1B and Figure S4 in Additional file 1).

To confirm that the KMTase activity of SETDB1 is critical for ERV silencing in mESCs [20], we analyzed the *Setdb1 *conditional KO mESC line, either unmodified (SETDB1 KO) or stably expressing wild-type (wt) (SETDB1 KO TG+) or KMTase-defective (SETDB1 KO C1243A) SETDB1 transgenes, the latter harbouring a single amino acid change in the catalytic domain [20]. As expected, robust derepression of ERVs is observed in the SETDB1 KO line (Figure 1C). Despite the fact that the SETDB1 C1243A line expresses an approximately threefold higher level of *Setdb1 *than wt cells (Figure 1D and [20]), derepression of several of these ERVs is also observed in this transgenic line, confirming that SETDB1 KMTase activity is essential for ERV silencing. Interestingly, the extent of derepression was dependent on the ERV family. The level of upregulation of MusD and IAPEz elements was equivalent in the SETDB1 KO and catalytic mutant lines, suggesting that silencing of these elements depends on the KMTase activity of SETDB1. MMERVK10C and GLN show a lower level of derepression in the SETDB1 C1243A line than the SETDB1 KO line, and MLV remains completely restricted in the SETDB1 C1243A line. Similar results were noted previously in Northern blot analyses [20]. Taken together, these results indicate that different ERV families are subject to SETDB1-mediated silencing generally dependent on SETDB1 catalytic activity.

### Depletion of HP1β but not HP1α leads to modest upregulation of select ERV families

**Figure 2:**
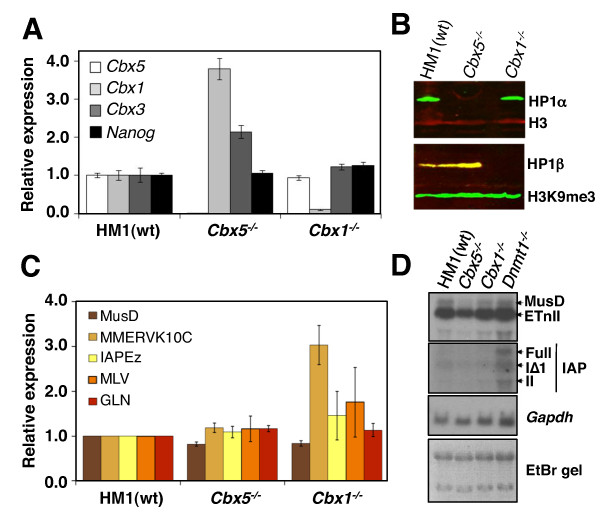
**Expression of heterochromatin protein 1 genes and ERVs in the *Cbx5^-/- ^*and *Cbx1^-/- ^*mESCs**. **(A) **qRT-PCR with primers specific for *Cbx5 *(encoding HP1α) *Cbx1 *(encoding HP1β), *Cbx3 *(encoding HP1γ) and the pluripotency factor *Nanog *in the ***Cbx5***^-/- ^and ***Cbx1***^-/- ^mESC lines confirms the KOs and reveals compensatory upregulation of the genes encoding the remaining HP1 proteins in the *Cbx5^-/- ^*line. Expression levels were normalized to *β-actin *relative to wt, and the data are presented as means ± SD for three technical replicates. **(B) **Western blot analysis of whole-cell lysates confirms the lack of expression of HP1α and HP1β in the *Cbx5*^-/- ^and *Cbx1*^-/- ^mESC lines, respectively. **(C) **Expression of representative ERV families in the HM1 (wt), *Cbx5*^-/- ^and *Cbx1*^-/- ^mESCs was determined by qRT-PCR. Expression levels were normalized to *β-actin *relative to wt. Data are presented as means ± SD of four independent experiments, each of which was performed in triplicate. **(D) **Northern blot analysis of RNA isolated from the parental HM1,*Cbx5*^-/- ^and *Cbx1*^-/- ^mESC lines using probes specific for ETnII, MusD and IAP ERVs are shown. RNA from *Dnmt1^-/- ^*mESCs, in which IAP elements are upregulated approximately fourfold [20,86] and MusD elements are upregulated approximately 1.5-fold [86], was used as a control.

Having confirmed that the KMTase activity of SETDB1 is required for efficient silencing of MMERVK10C, MusD and IAPEz, we next sought to determine whether the archetypal heterochromatic H3K9me2/3 readers HP1α and HP1β [40], both of which are enriched on IAPEz, MusD and MLV ERVs [20], are the effectors of transcriptional suppression of these elements. We generated *Cbx5 *(HP1α) KO mESCs (Figure S1 in Additional file 1) and *Cbx1 *(HP1β) KO mESCs (Figure S2 in Additional file 1) and confirmed downregulation of the corresponding genes at the mRNA level by qRT-PCR and at the protein level by Western blot analysis. Equivalent levels of expression of the pluripotency factor *Nanog *were detected in these lines, indicating that deletion of HP1 proteins does not stimulate differentiation (Figure 2A). Interestingly, while compensatory upregulation of the *Cbx1 *and *Cbx3 *genes was observed at the mRNA level in the *Cbx5*^-/- ^line, upregulation of these genes was not observed at the protein level (Figure 2B).

Surprisingly, unlike deletion of *Setdb1*, deletion of *Cbx5 *does not lead to upregulation of any members of the ERV families analyzed, as determined by qRT-PCR (Figure 2C) or Northern blot analysis (Figure 2D). Similarly, deletion of *Cbx1 *has no effect on MusD, MLV or GLN elements. Although *Cbx1 *deletion does result in modest derepression of MMERVK10C (approximately 3-fold) and IAPEz (approximately 1.5-fold) relative to the parental HM1 line, these ERVs show approximately 47-fold and approximately 3-fold upregulation respectively, in the *Setdb1 *KO line, relative to the parental TT2 line, (see Figure 1). Taken together, these results indicate that in contrast to SETDB1, HP1α and HP1β play no role or a relatively minor role, respectively, in class II ERV silencing in mESCs.

### Depletion of HP1α results in a modest reduction of DNA methylation at IAPEz ERVs

**Figure 3:**
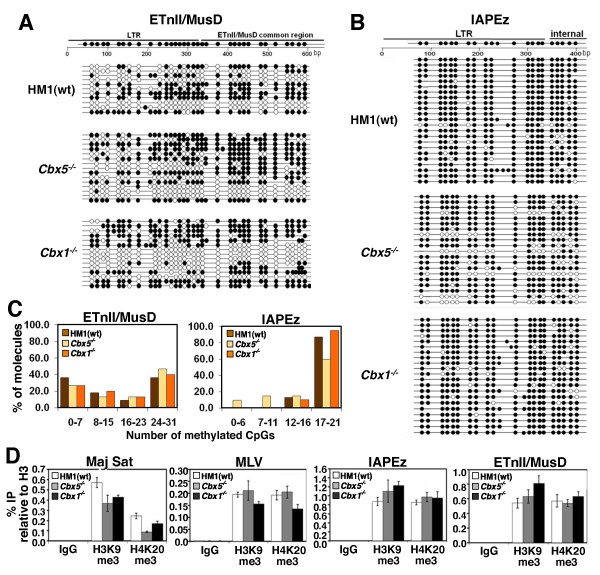
**DNA methylation and chromatin marks at ERVs in *Cbx5^-/- ^*and *Cbx1^-/- ^*mESCs**. **(A) **An approximately 600-bp fragment of the LTR and downstream region of ETnII/MusD ERVs was analyzed by bisulphite sequencing using primers that detect 105 ETnII/MusD elements, according to *in silico *PCR analysis (UCSC Genome Browser, http://genome.ucsc.edu/). **(B) **An approximately 400-bp fragment of the LTR and downstream region of IAP ERVs was analyzed by bisulphite sequencing using primers that detect 1,461 IAP elements. **(C) **Bisulphite-sequenced molecules were binned into four categories, depending on the number of methylated CpG sites detected, and the data are presented as the percentage of all clones for each cell line in each bin. While the ETnII/MusD family shows no difference in DNA methylation, several IAP molecules in the *Cbx5^-/- ^*cell line exhibit partial demethylation. **(D) **Native ChIP (N-ChIP) with antibodies against H3K9me3, H4K20me3 and pan-H3 was followed by qPCR using primers specific for major satellite repeats and IAP, MLV and MusD ERVs, and the data are presented as means ± SD for three technical replicates. The level of H4K20me3 was reduced by more than 50% at major satellite repeats in the *Cbx5^-/- ^*mESCs but remained at the same level at ERVs.

We recently demonstrated that while G9a is dispensable for silencing of ERVs, this H3K9 KMTase is required for efficient DNA methylation of these elements in mESCs [83]. Similarly, DNA methylation of major satellite repeats is dependent upon the H3K9 KMTase SUV39H1/2 in mESCs [84]. Intriguingly, HP1 proteins are required for DNA methylation of repetitive elements in *Neurospora *[48,85], but the role of HP1 proteins in DNA methylation of ERVs in mESCs has not been explored. To address this question, ETnII/MusD and IAPEz families, shown previously to be densely DNA methylated in mESCs [20,83,86], were analyzed by bisulphite sequencing using genomic DNA isolated from wt, *Cbx1*^-/- ^and *Cbx5^-/- ^*mESCs. In wt cells, several copies of ETnII and MusD were either completely unmethylated or hypomethylated specifically at the 5' end of the LTR (Figures 3A and 3C) as observed previously [86]. The number of methylated CpG sites per element of this family remained similar in either of the *Cbx *KO lines. In contrast, while the level of DNA methylation was very high at IAPEz elements in wt cells, several IAP molecules showed reduced levels of DNA methylation in the *Cbx5*^-/- ^cell line (Figures 3B and 3C), indicating that HP1α plays a role in DNA methylation of a subset of IAP elements, presumably dependent upon their genomic location. Nevertheless, as discussed above, this modest decrease in DNA methylation did not result in derepression of IAP elements in these cells.

### Neither HP1α nor HP1β are essential for H4K20me3 deposition at ERVs

Although H4K20me3 is dispensable for proviral silencing in mESCs, its deposition by SUV4-20H at ERVs requires SETDB1-deposited H3K9me3 [20]. On the basis of the fact that in mouse embryonic fibroblasts (MEFs), H4K20me3 at satellite repeats is dependent on SUV39H1/2-deposited H3K9me3 and subsequent binding of HP1 to this mark [82], we investigated whether H4K20me3 at ERVs is also dependent upon HP1 proteins in mESCs. Native ChIP (N-ChIP) followed by qPCR revealed that H4K20me3 enrichment was reduced by more than 50% at major satellite repeats in the *Cbx5^-/- ^*line (Figure 3D), demonstrating that as in MEFs [82], HP1 proteins are required for efficient H4K20me3 deposition at pericentric heterochromatin in mESCs. However, this mark is not entirely lost in either of the KO lines, presumably due to partial redundancy of HP1 proteins at major satellites. In line with these findings, it was recently shown that HP1β is dispensable for H4K20me3 and H3K9me3 deposition and localization in heterochromatin of mouse neurons [81]. Similarly, H4K20me3 levels at IAPEz, ETnII/MusD and MLV ERVs in the *Cbx1*^-/- ^and *Cbx5*^-/- ^lines remained at levels similar to the wt parent line, demonstrating either that H4K20me3 is deposited independently of HP1 binding or that these proteins act redundantly to promote deposition of H4K20me3 at these elements. As expected, H3K9me3 also remained unaltered in the absence of HP1α or HP1β (Figure 3D).

### HP1β plays a role in the spreading of H4K20me3 but not H3K9me3 from ERVs into flanking genomic regions

**Figure 4:**
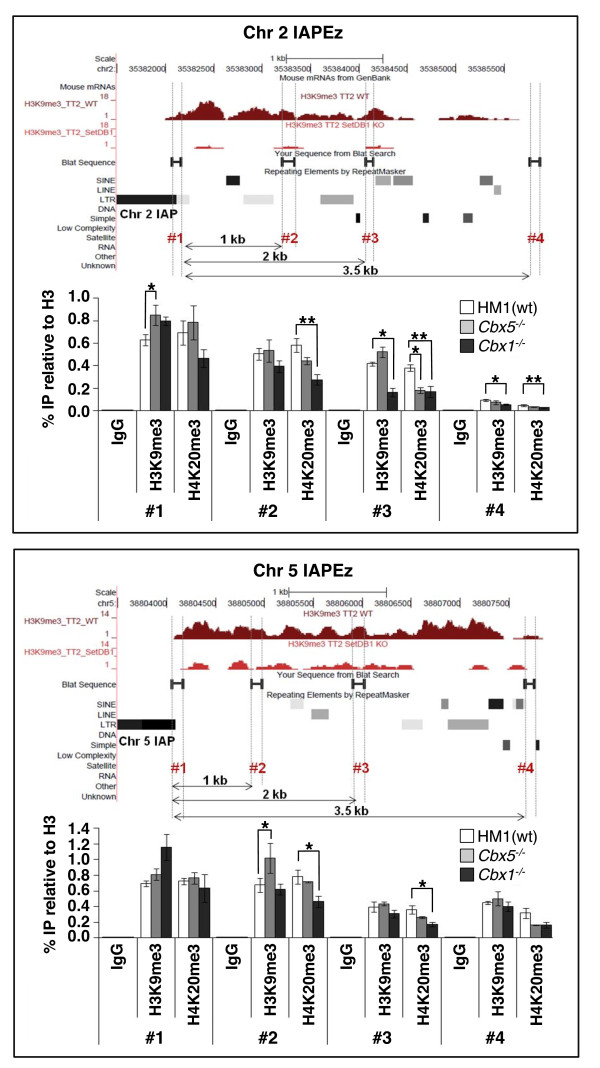
**HP1β plays a role in H4K20me3 but not H3K9me3 spreading from ERVs into flanking genomic DNA**. N-ChIP was performed with H3K9me3-, H4K20me3- and pan-H3-specific antibodies using chromatin isolated from HM1, *Cbx5^-/- ^*and *Cbx1^-/- ^*mESCs. The level of enrichment of these modifications at the flanks of two full-length IAP elements on chromosomes 2 and 5 as well as at positions approximately 1 kb, 2 kb and 3.5 kb distal to these flanking regions, was determined by qPCR. Data are presented as means ± SD of three technical replicates, and pairs of control and experimental samples with *P < 0.05 and **P < 0.01 (two-tailed Student's *t*-test) are shown. H3K9me3 enrichment levels across these genomic regions as determined using our previously published ChIP-seq data sets [19] are also shown for wt and *Setdb1 *KO mESCs.

Intriguingly, while HP1 homologs play a positive role in heterochromatin spreading in *Drosophila *[62,63] and mammals [42,57], HP1 plays a critical role in inhibiting aberrant spreading of heterochromatin in *Neurospora *[69]. Genome-wide analysis of H3K9me3 in wt mESCs reveals high levels of H3K9me3 in the immediate flanks of ERVs, including IAPEz, MusD and MLV elements, with progressively lower levels of this mark at distances farther from the ERV integration site [18,19]. As expected, deposition of H3K9me3 in these regions is SETDB1-dependent [19] (see Figure 1B and Figure S4 in Additional file 1). Notably, the profile of H4K20me3 in the genomic regions flanking IAP, MusD and MLV elements is similar to that of H3K9me3 and the relative levels of both marks are consistent with their abundance in each ERV family (that is, IAP > MusD > MLV [20]). To determine whether spreading of H3K9me3 and/or H4K20me3 is affected in *Cbx1*^-/- ^and/or *Cbx5*^-/- ^mESCs, we examined these marks at the flanks of two randomly chosen full-length IAPEz elements and three genomic locations distal to the integration sites of these ERVs by ChIP-qPCR (Figure 4). IAPEz elements were chosen because, among the ERVs analyzed, on average, this family showed the highest mean H3K9me3 density in flanking genomic regions (Figure 1B and Figure S4 in Additional file 1). As expected, in wt cells, the levels of both H3K9me3 and H4K20me3 generally declined as the distance from the IAP increased, dropping substantially at approximately 3.5 kb. Depletion of either HP1 protein did not show a consistent effect on the spreading of H3K9me3 into the flanks of the selected IAP elements, since at the majority of regions surveyed enrichment was not statistically significantly different in each of the KO lines from the wt control. HP1β may be involved in propagation of H3K9me3 beyond 2 kb from the IAP assayed on chromosome 2, however, suggesting that at least at some loci, HP1β may facilitate the spreading of H3K9me3.

Analysis of H4K20me3 in the same regions revealed no decrease in this mark in the *Cbx5*^-/- ^line. In contrast, relative to the HM1 parent line, the *Cbx1*^-/- ^line showed a consistent, approximately 1.5- to 2-fold decrease (*P *< 0.05, two-tailed Student's *t-*test) in H4K20me3 at both loci in distal regions 2 and 3 (Figure 4). Thus, while neither HP1 protein is required for deposition of H4K20me3 at the ERVs themselves (see Figure 3D), HP1β may generally be involved in the spreading of this covalent mark into the genomic regions flanking these repetitive elements.

### Application of novel ERV reporter lines in a siRNA-based screen of H3K9me3-binding proteins

**Figure 5:**
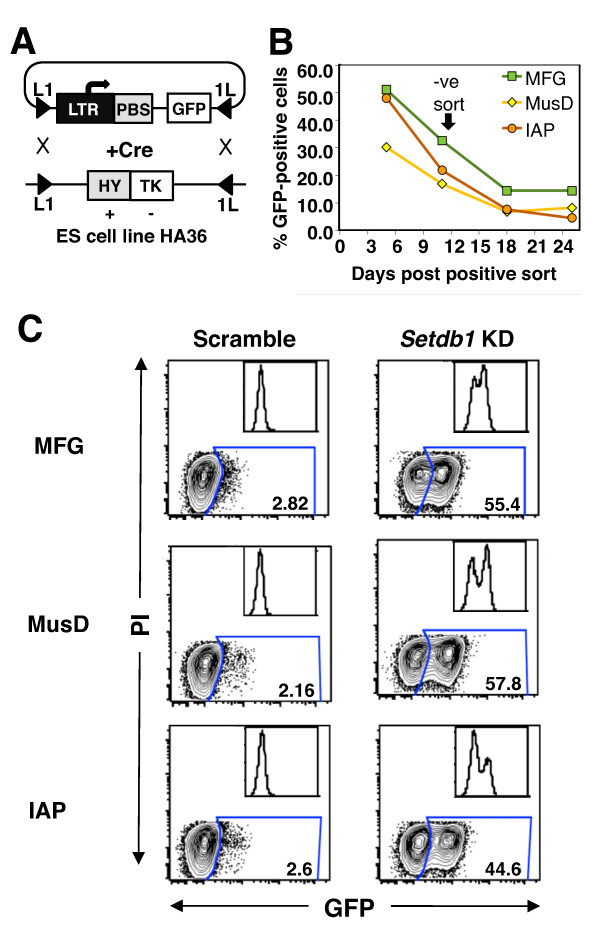
**Silencing kinetics and reactivation of ERV reporters integrated in a specific genomic site**. **(A) **Scheme for targeting of ERV reporters into a specific genomic site in mESCs via recombinase-mediated cassette exchange (RMCE). The mESC line HA36 contains a hygromycin B and herpes simplex virus thymidine kinase (HyTK) cassette between inverted Lox sites (L1 and 1L). MFG-green fluorescent protein (*GFP*), MusD-*GFP *and IAP-*GFP *proviral reporter cassettes, which contain the Moloney murine leukaemia virus, MusD (approximately +130 bp of downstream sequence) and IAP (approximately +450 bp of downstream sequence) LTRs, respectively, flanked by L1 and 1L sites, were cotransfected into the HA36 line with a Cre recombinase expression vector. Negative selection with ganciclovir eliminated cells with the original HyTK cassette, yielding pools of cells harbouring the proviral reporter cassettes predominantly integrated at the same site. **(B) **The kinetics of silencing of the MFG, MusD and IAP cassettes after reactivation of the RMCE pool with siRNA against *Setdb1 *are shown. **(C) **GFP-negative cells were sorted at day 12 postinduction with *Setdb1 *siRNA. Robust reactivation of GFP **} **expression from each of these pools of cells was observed upon secondary *Setdb1 *knockdown (KD). Flow cytometry data are presented as contour plots and histograms of 10,000 viable (propidium iodide (PI)-negative) cells.

In addition to HP1 proteins, a number of other chromodomain proteins, including CDYL2, CBX2, CBX4, CBX7 and MPP8, as well as the Tudor domain-containing protein TDRD7, were recently shown to bind H3K9me3 *in vitro *[71-73,75,76]. To address whether any of the H3K9me3 readers expressed in mESCs (all of those mentioned above with the exception of *Cbx4 *and *Tdrd7*) play a role in SETDB1-dependent silencing, we used recombinase-mediated cassette exchange (RMCE) [87,88] (Figure 5A) to derive novel mESC lines with single-copy proviral reporters integrated at a specific genomic site. Specifically, constructs harbouring the green fluorescent protein (*GFP*) gene downstream of the MusD or IAP LTR promoters were generated and introduced into the same genomic site in the mESC line HA36 (a gift from F Lienert and D Schübeler) via RMCE. In parallel, the MFG-*GFP *construct [89] derived from the Moloney murine leukaemia virus (MMLV) and efficiently silenced in mESCs and embryonic carcinoma cells [20,90-93], and a cytomegalovirus (CMV)-*GFP *cassette were introduced into the same site. Following Cre-mediated recombination and a five-day negative selection with ganciclovir to exclude cells harbouring the original hygromycin B-herpes simplex virus thymidine kinase fusion (HyTK) cassette, each of the LTR reporters became silenced, while the CMV promoter maintained expression (data not shown). To select cells that contain the ERV-driven *GFP *gene silenced via the SETDB1 pathway, we transiently transfected the GFP-negative ganciclovir-resistant pools with siRNA specific for *Setdb1*. Depending on the cassette, GFP expression was induced in 20% to 65% of viable cells and these GFP-positive cells were isolated by fluorescence-activated cell sorting (FACS). The LTR reporter cassettes were progressively resilenced over approximately three weeks in culture (Figure 5B), and the negative populations were sorted at day 12 to be used as reporters. Importantly, GFP was efficiently reactivated in each population upon subsequent treatment of these pools with *Setdb1 *siRNA (Figure 5C), confirming that silencing of these LTR reporters is SETDB1-dependent at this integration site.

**Figure 6:**
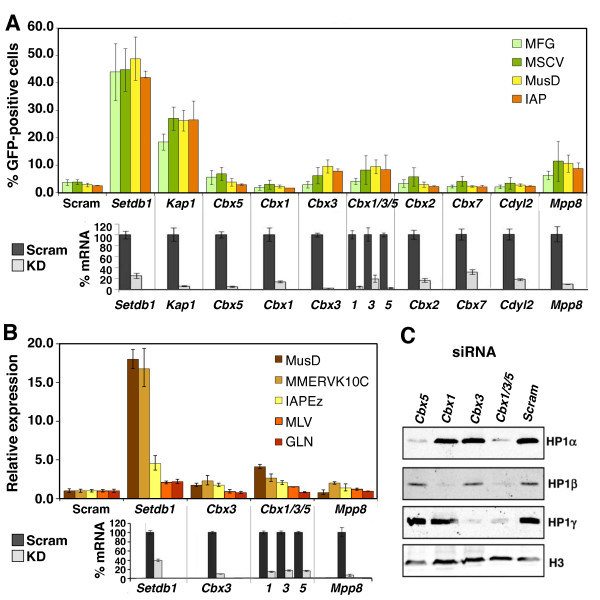
**Reactivation of ERV reporters and ERVs upon siRNA-mediated KD of H3K9me3-binding proteins**. **(A) **The percentage of enhanced green fluorescent protein-positive mESCs with reactivated ERV reporters was determined by flow cytometry (upper panel) on day 5 after the second transfection with siRNA against specified H3K9me3 readers. At least 10,000 cells were collected for each sample. Data are presented as means ± SD of three biological replicates. KD efficiency was determined by qRT-PCR at 30 hours after the second siRNA transfection (lower panel). Data are presented as means ± SD of three technical replicates. **(B) **Relative expression of ERVs at day 5 after the second KD with the indicated siRNA pool (upper panel), along with the efficiency of each KD, as determined by qRT-PCR at 30 hours after the second siRNA transfection (lower panel) is presented as means ± SD of three technical replicates. For each amplicon, expression was normalized to *β-actin *relative to scramble siRNA KD. **(C) **Western blot analysis of HP1 proteins in single- and triple-KD cells at day 5 after the second siRNA transfection is shown. H3 was used as a loading control.

To determine whether any of the remaining chromodomain-containing H3K9me3 readers expressed in mESCs are required for SETDB1-mediated silencing, we knocked down *Cbx3 *(HP1γ) as well as *Cdyl2*, *Cbx1*, *Cbx2*, *Cbx5*, *Cbx7 *and *Mpp8 *in the above-described reporter lines and a previously described pool of mESCs harbouring the silent murine stem cell virus (MSCV) provirus [20]. As expected, treatment of the MFG, MSCV, IAP and MusD reporter lines with *Setdb1 *and *Kap1 *specific siRNAs induced GFP expression in approximately 45% and approximately 25% of cells, respectively (Figure 6A, upper panel). In contrast, knockdown (KD) of each of the H3K9me3-binding proteins failed to induce GFP expression to the levels seen upon KD of *Setdb1 *or *Kap1*, despite efficient depletion of the target mRNAs (Figure 6A, lower panel). KD of genes encoding other chromodomain-encoding proteins with H3K9me-binding properties, such as *Cdyl *[72] and *Chd4 *[94,95], also did not result in reporter reactivation (Figure S5 in Additional file 1).

KD of *Cbx3 *and *Mpp8 *did induce GFP expression in about 10% of treated cells, raising the possibility that these H3K9me3 readers act in a redundant manner to maintain these ERV reporters in a silent state. However, simultaneous KD of *Cbx3 *in combination with *Mpp8 *(Figure S6 in Additional file 1) or of *Cbx3 *in combination with *Cbx1 *and *Cbx5 *(*Cbx1/3/5*) (Figure 6A) did not significantly increase the percentage of GFP-positive cells over that observed with individual KD, despite efficient depletion of each mRNA. KD of *Cbx1 *and *Cbx3 *in the *Cbx5^-/- ^*mESCs and KD of *Cbx3 *and *Cbx5 *in the *Cbx1^-/- ^*mESCs showed similar results (data not shown). Thus, none of the assayed chromodomain-encoding proteins with H3K9-binding activity are essential for proviral silencing.

The H3K4me2/3 demethylase JARID1C (SMCX, which does not harbour a chromodomain, is capable of binding H3K9me3 via its plant homeodomain (PHD) [96], and its yeast homologue, Lid2, interacts directly with the H3K9 HMTase Clr4 [97]. These interactions suggest that JARID1C may direct H3K4 demethylation to loci marked by H3K9me3, promoting silencing. However, KD of the *Jarid1 *genes expressed in mESCs, including *Jarid1a*, *Jarid1b *and *Jarid1c*, either alone or in combination, leads to only modest reactivation of the proviral reporters, indicating that H3K9me3-recognizing H3K4 demethylases are not critical for maintenance of ERV silencing (Figure S7 in Additional file 1). Similarly, KD of *Uhrf1 *(NP95 in mouse and ICBP90 in human), which was recently shown to bind H3K9me3 via its PHD or SRA (SET- and RING-associated) domain [79,98-100], and/or KD of a related gene, *Uhrf2 *yields minimal upregulation of the four ERV reporters (Figure S8 in Additional file 1). Based on its pericentric localization [98], the main function of ICBP90 may lie in replication of heterochromatin and transcriptional regulation of major satellites [101], which show SUV39H1/2-dependent H3K9me3. Taken together, these data reveal that none of the known H3K9me3 readers are essential for silencing of ERVs that are repressed by the SETDB1 pathway.

To determine whether *Cbx3 *and *Mpp8*, the H3K9me3 readers which showed the highest reactivation of the LTR reporters, are required for silencing of ERVs, we performed qRT-PCR on cDNA isolated from wt TT2 and siRNA-treated mESCs. In *Setdb1 *KD cells, the MMERVK10C and MusD families showed the highest level of derepression, as expected. The same families, however, are only modestly upregulated upon KD of *Cbx3 *or *Mpp8 *(Figure 6B, upper panel), despite reduction of the target mRNA to 9% to 30% of wt levels (Figure 6B, lower panel) and dramatic downregulation of *Cbx3 *at the protein level, as determined by Western blot analysis (Figure 6C).

Finally, to determine whether HP1 proteins act redundantly to silence ERVs, we performed simultaneous KD of *Cbx1*, *Cbx3 and Cbx5*. Strikingly we observed only modest reactivation of each of the ERVs analyzed (Figure 6B, upper panel). Similar levels of upregulation of MMERVK10C and IAPEz ERVs in the *Cbx1*^-/- ^mESCs (3.0- and 1.5-fold, respectively) (see Figure 2C), the *Cbx3 *KD (2.3- and 1.8-fold, respectively) and the triple *Cbx1/3/5 *KD (2.6- and 2.1-fold, respectively) suggest that *Cbx1 *and *Cbx3 *account for most of the HP1-mediated silencing of these ERV families. However, MusD elements, which are upregulated approximately fourfold in the triple KD, were not upregulated in any of the KOs, suggesting that all three HP1 proteins must be depleted to generate the relatively modest level of derepression observed for this family. Although we cannot rule out the possibility that an insufficient level or duration of HP1 depletion upon KD is responsible for these negative results, Western blot analysis revealed almost complete loss of all HP1 proteins in cells simultaneously depleted of *Cbx1*, *Cbx3 *and *Cbx5 *(Figure 6C). On the basis of the lack of ERV upregulation upon simultaneous KD of all three HP1 isoforms, we postulate that redundancy in HP1 function might not be the major factor preventing broad ERV reactivation. Similarly, as the maximum level of ERV reactivation upon KD of the remaining H3K9me3 readers is considerably lower than that observed in *Setdb1 *KO or *Setdb1 *KD cells, we conclude that none of these H3K9me3 readers play a major role in SETDB1-mediated ERV silencing in mESCs.

## Discussion

### Role of H3K9me3 and HP1 in silencing of ERVs

We and others have recently shown that the H3K9me3 KMTase SETDB1 is critical for silencing of class I and class II ERVs in mESCs [20,21]. However, the mechanism by which H3K9me3 modification leads to their transcriptional repression is currently unclear. In the present study, we have shown that HP1α plays a modest role in maintaining DNA methylation of IAPEz ERVs, while HP1β plays a modest role in promoting the spreading of H4K20me3 into the regions flanking these elements. HP1β also contributes to silencing of select IAPEz and MMERVK10C elements, but has no effect on DNA methylation of the ERVs analyzed. However, individual depletion of HP1α, HP1β and all other candidate H3K9me3 readers does not result in upregulation of ERVs or ERV reporters to a level observed in *Setdb1 *KO or *Setdb1 *KD mESCs, indicating that these factors either play only a modest role in silencing or act redundantly in this process. Strikingly, robust proviral derepression was not observed, even after simultaneous depletion of all three HP1 proteins, ruling out the latter, at least for these readers. Nevertheless, we cannot exclude the possibility that an as yet unidentified H3K9me3-binding protein and/or functional redundancy between H3K9me readers other than HP1 proteins may be required for H3K9me3-mediated ERV repression.

### H3K9me3-dependent, H3K9me3 reader-independent proviral silencing?

Consistent with our observation that HP1s do not play a major role in transcriptional silencing of ERVs in mESCs, tethering of HP1 proteins in *Drosophila *inactivates only a limited number of reporter lines [102,103]. Indeed, H3K9me3-dependent silencing may occur through mechanisms independent of H3K9me3 readers, such as by preventing the binding of transcription factors essential for transcription and/or the recruitment of the RNA polymerase II complex itself.

Specifically, H3K9me3 may directly or indirectly inhibit the deposition of active covalent histone marks. Acetylation of H3 at lysine 9 (H3K9Ac), for example, which is incompatible with methylation at this residue, promotes recruitment of chromatin remodelers and binding of RNA polymerase II in promoter regions [104-108], and the histone acetyltransferases GCN5 and PCAF, which acetylate H3K9 [109,110], are required for expression of specific genes [110] and retroviral elements [111]. Furthermore, hyperacetylation of H3 and H4 occurs concomitantly with IAP upregulation in MEFs and early embryos deficient in lymphoid-specific helicase (LSH) [112], implicating histone acetylation in ERV transcriptional activity. Intriguingly, in *Xenopus *oocytes expressing human H3K9 KMTases and HP1, H3K9me3 mediates transcriptional repression independently of HP1 recruitment through a mechanism that involves histone deacetylation [59].

Alternatively (or in addition), H3K9me3 may block transcription by indirectly inhibiting phosphorylation at serine 10 (H3S10ph) in the proviral promoter region. Intriguingly, transcriptional activation of the mouse mammary tumour retrovirus is dependent on H3S10ph and hyperacetylation of H3, mediated by binding of the nuclear factor 1 (NF-1) transcription factor to the proviral LTR [113-115]. Predicted NF-1-binding sites are also found in the LTRs of other ERVs, including ETn elements [116], implicating a broad role for H3S10ph in transcription of these elements. While experiments directly addressing whether H3K9me3 blocks phosphorylation of H3S10ph have not been conducted in mammalian cells, H3K9me3 severely inhibits H3S10ph mediated by the Ipl1/aurora kinase in yeast [117].

Finally, H3K4 di- and trimethylation, marks also associated with the promoter regions of transcriptionally active genes, may also be inhibited by the presence of H3K9me3. Indeed, the H3K4 methyltransferases ASH1L [118] and SET7 [119] are less efficient in depositing H3K4me on histones marked with H3K9me in human cell lines, and H3K9me3 and H3K4me3 are mutually exclusive marks in mESCs [18]. While H3K4me2 is detected in the promoter region of IAP elements in *Lsh^-/- ^*MEFs concomitant with their upregulation [120], the appearance of such active marks may be a consequence of, rather than a prerequisite for, transcriptional activation. To directly address the role of H3K4 methylation in retroviral expression, we sought to determine whether KD of *Wdr5*, a subunit of MLL/SET1 H3K4 methyltransferase complexes, inhibits *Setdb1 *KD-induced activation of the ERV reporters. We found that simultaneous KD of *Setdb1 *and *Wdr5*, reduced the level of reactivation of all ERV reporters, especially MusD and IAP (Figure S9 in Additional file 1), indicating that H3K4me3, the catalytic product of WDR5-containing complexes [121], is indeed required for optimal transcription of ERVs. Thus, the presence of H3K9me3 may effectively block transcription by inhibiting deposition of H3K4me3 and/or the other active marks mentioned above.

### Heterochromatin spreading into sequences flanking ERV

Heterochromatin spreading is thought to involve a reiterative process of HP1 proteins binding to H3K9me2/3 [36,37] followed by the recruitment of protein complexes with H3K9me2/3 catalytic activity, such as SUV39H1/2 [65] and SETDB1 [61]. Consistent with this model, HP1 proteins have been implicated in heterochromatin spreading in *Drosophila *[62,63], yeast [64] and mammals [42,57]. Moreover, H3K9me3, a hallmark of silent chromatin, is abundant in the vicinity of ERVs [18,19]. However, our results indicate that HP1α and HP1β play only a modest role, if any, in the spreading of H3K9me3 into the sequences flanking ERVs. In contrast, HP1β is required for efficient spreading of H4K20me3 at the IAP ERVs analyzed. Although the biological role of H4K20me3 spreading is still unclear, recent studies have indicated that this covalent mark is involved in the maintenance of genomic stability [122-124]. Intriguingly, a role for HP1 in the DNA damage response independent of H3K9me3 has also been reported [125,126]. The availability of HP1β KO embryos will allow for studies aimed at addressing whether the distribution of H4K20me3 is dependent upon this protein and whether DNA damage repair pathways are perturbed *in vivo*.

## Conclusions

In this work, we demonstrate the surprising finding that despite the accepted function of HP1 proteins in H3K9me-dependent gene silencing and the critical role of H3K9me3 in transcriptional repression of class I and class II ERVs, HP1α and HP1β are not required for silencing of these repetitive elements. Furthermore, while neither HP1α nor HP1β is essential for DNA methylation or the deposition of H3K9me3 or H4K20me3 within ERVs, HP1β plays a role in the spreading of the latter into sequences flanking these elements. Using a RNAi-based screen with newly derived mESCs harbouring novel ERV reporters, we have shown that the remaining proteins reported to bind H3K9me3 *in vitro*, including HP1γ, CDYL, CDYL2, CBX2, CBX7, MPP8, UHRF1 and JARID1A-C, are also dispensable for ERV silencing. The lack of proviral derepression in these experiments may be explained by functional redundancy of these or as yet unidentified H3K9me3 readers. Alternatively, H3K9me3 may repress ERV transcription via inhibiting deposition of covalent histone modifications required for transcription. The ERV reporter cell lines generated here should be useful in future screens of factors predicted to play a role in proviral expression. Regardless, additional studies aimed at delineating the functional significance of H3K9 readers, including nuclear processes not directly related to transcription, are clearly warranted.

## Materials and methods

### Cell culture, constructs and recombinase-mediated cassette exchange

To produce the *Cbx5^-/- ^*and *Cbx1 *mESC lines, each *Cbx *allele was targeted sequentially using two different targeting vectors. See Figures S1 and S2 in Additional file 1 for a detailed description of the ESC-KO derivation. mESCs were cultured in DMEM supplemented with 15% fetal bovine serum (HyClone Laboratories, Logan, UT, USA), 20 mM 4-(2-hydroxyethyl)-1-piperazineethanesulfonic acid, 0.1 mM nonessential amino acids, 0.1 mM 2-mercaptoethanol, 100 U/mL penicillin, 0.05 mM streptomycin, leukaemia-inhibitory factor and 2 mM L-glutamine on gelatinized plates. For RMCE into HA36 cells, CMV was cut out of the L1-CMV-GFP-1L vector [127] by restriction with *Cla*I and *Nhe*I restriction enzymes. IAP and MusD LTR, together with the downstream sequence, were cloned into the resulting *Cla*I*-Nhe*I site upstream of the enhanced green fluorescent protein (*EGFP*) gene. MusD from the C57BL/6 genomic DNA on chr8:131270355-131277831 (*mm9*) was cloned, an element similar in sequence to those commonly expressed in wt cells [20]. The *Nhe*I site at nt 444 prevented us from including a longer fragment. However, this sequence still included the 319 bp 5'-LTR and 125 bp immediately downstream of it. A fragment containing a LTR and a downstream sequence, approximately 800 bp in total, was cloned for an IAP reporter. The element chosen was the one at the site of a novel insertion into the A/WySn mouse strain [128,129] and was cloned from the DNA of the respective strain. All inserts were confirmed by sequencing. The primer sequences are given in Table S1 in Additional file 1.

### Recombinase-mediated cassette exchange, transfection and transgene selection

For targeting of the ERV reporter constructs into the genome, Cre RMCE was used [87,130]. The HA36 mESC line contains a cassette with the *HyTK *fusion gene at the random integration site which allows CMV-GFP expression for multiple passages (cell line a gift from F Lienert and D Schübeler). This selectable marker allows for positive selection through resistance to hygromycin B and for negative selection through sensitivity to ganciclovir. HA36 mESCs were cultured in 25 μg/mL hygromycin B for 14 days before transfection to select for cells expressing the fusion gene. Cells were transfected with Lipofectamine 2000 (Invitrogen, Carlsbad, CA, USA) in a 24-well plate according to the manufacturer's recommendations. Briefly, 1.5 μg of a cassette with a MusD, IAP or MFG insert was cotransfected with 0.5 μg of CMV-Cre plasmid using 2 μL of Lipofectamine 2000 per well. After three days, cells were transferred to medium containing 3 μM ganciclovir to select against cells still expressing the *HyTK *fusion gene. Cells were grown in ganciclovir-containing medium for five or more days, with subculturing performed when necessary.

### siRNA-mediated knockdown

For reporter assays, 10,000 mESCs per well of a 96-well plate were seeded into antibiotic-free mESC medium the day before transfection. Transfection was performed according to the manufacturer's protocol using 100 nM concentrations of each siRNA (siGENOME SMARTpool reagent Dharmacon, Lafayette, CO, USA) and 0.4 μL of DharmaFECT 1 siRNA transfection reagent (Dharmacon) per well. On the first day after transfection, approximately 1/5^th ^of the cells were transferred into another 96-well plate containing antibiotic-free mESC medium, and the KD was repeated on the third day. The next day, approximately 1/2 of the cells were transferred into a 24-well or 12-well plate, and flow cytometry was performed on day 4 or 5 after the second KD. For RNA or protein collection, the first KD was performed in a 12-well plate and the cells were transferred to two 6-cm dishes the next day. The day after the second KD in 6-cm dishes, three-fourths of the cells were collected for RNA for confirmation of KD efficiency, and the rest were plated onto two 10-cm dishes for expansion and collection for RNA or protein on day 4 after the second KD.

### Preparation of genomic DNA, bisulphite treatment, T/A cloning and sequencing

Genomic DNA was extracted using DNAzol reagent (Invitrogen), and bisulphite conversion of DNA was performed using the EZ DNA Methylation Kit (Zymo Research, Orange, CA, USA) according to the manufacturer's protocol. The approximately 370 bp of IAP and approximately 590 bp of ETnII/MusD element sequence containing the LTR and the downstream region were amplified from converted DNA by PCR using Platinum *Taq *(Invitrogen). The primer sequences are given in Additional file 1, Table S1. PCR products from three separate PCRs for each sample were cloned using the pGEM-T Easy Vector System kit (Promega, Madison, WI, USA). All sequences had a conversion rate of > 98%. QUMA http://quma.cdb.riken.jp/top/index.html, with some follow-up processing, was used for analysis of bisulphite data [131].

### Native chromatin immunoprecipitation assay and quantitative PCR

Briefly, 1 × 10^7 ^mESCs **} **for each cell line were resuspended in douncing buffer and homogenized through a 25-gauge 5/8-inchneedle syringe for 20 repetitions. A quantity of 1.875 μL of 20 U/μL micrococcal nuclease (MNase; Worthington Biochemical Corp., Lakewood, NJ, USA) was added and incubated at 37°C for 7 minutes. The reaction was quenched with 0.5 M ethylenediaminetetraacetic acid and incubated on ice for 5 minutes; then 1 mL of hypotonic buffer was added and incubated on ice for 1 hour. Cellular debris was pelleted, and the supernatant was recovered. Protein A/G Sepharose beads were blocked with single-stranded salmon sperm DNA and BSA, washed and resuspended in immunoprecipitation buffer. Blocked protein A/G Sepharose beads were added to the digested chromatin fractions and rotated at 4°C for 2 hours to preclear chromatin. A quantity of 100 μL of the precleared chromatin was purified by phenol-chloroform extraction, and DNA fragment sizes were analyzed and confirmed to correspond to one to three nucleosome fragments. Chromatin was subdivided into aliquots for each immunoprecipitated sample. Antibodies specific for unmodified H3 (H9289; Sigma-Aldrich, St Louis, MO, USA), H3K9me3 (Active Motif 39161, Carlsbad, CA, USA), H4K20me3 (Active Motif 39180) and control immunoglobulin G (I8140; Sigma-Aldrich, St Louis, MO, USA) were added to each tube and rotated at 4°C for 1 hour. The antibody-protein-DNA complex was precipitated by adding 20 μL of the blocked protein A/G Sepharose beads and rotated at 4°C overnight. The complex was washed and eluted, and immunoprecipitated material was purified using the QIAquick PCR Purification Kit (Qiagen, Germantown, MD, USA). The purified DNA was analyzed by qPCR with respect to input using EvaGreen dye (Biotium, Hayward, CA, USA) and Maxima Hot Start *Taq *DNA Polymerase (Fermentas, Vilnius, Lithuania). Primers are listed in Table S1 in Additional file 1.

### RNA isolation, reverse transcription and quantitative RT-PCR

RNA was isolated using GenElute™ Mammalian Total RNA Miniprep Kit (Sigma-Aldrich) and reverse-transcribed using SuperScript III Reverse Transcriptase (Invitrogen) as per the manufacturers' instructions. Quantitative RT-PCR was carried out using SsoFAST™ EvaGreen Supermix (Bio-Rad Laboratories, Hercules, CA, USA) on StepOne™ version 2.1 software (Applied Biosystems, Foster City, CA, USA) in a total volume of 20 μL. Data are presented as means ± standard deviations of three technical replicates. Primer efficiencies were around 100%. Dissociation curve analysis was performed after the end of the PCR to confirm the presence of a single and specific product.

### Whole-cell protein extracts and Western blot analysis

Briefly, cells were resuspended in 2 ± Laemmli buffer and incubated at 100°C for 10 minutes. Cells were then homogenized through a 25-gauge needle syringe 10 to 15 times. Extracts were run on SDS-PAGE gels and transferred onto a membrane. Primary antibodies used were α-HP1α (05-684, 1:200 dilution; Upstate Biotechnology, Lake Placid, NY, USA), α-HP1β (MCA 1946, 1:100 dilution; AbD Serotec, Burlington, ON, Canada) and α-H3 (Active Motif 39163, 1:200 dilution, Carlsbad, CA, USA). Secondary antibodies used at 1:10,000 dilutions were IRdye 800CW (926-32210) and IRdye 680 (926-32221), both from LI-COR Biosciences (Lincoln, NE, USA). Membranes were analyzed using the Odyssey Infrared Imaging System (LI-COR Biosciences).

### Northern blot analysis

For each lane, 6 mg of RNA were denatured, electrophoresed in 1% agarose/3.7% formaldehyde gel in 1 ± 3-(N-morpholino)propanesulfonic acid buffer, transferred overnight onto a Zeta-Probe nylon membrane (Bio-Rad Laboratories, Hercules, CA, USA) and baked at 80°C. ETnII/MusD-, IAP- and *Gapdh*-specific probes were synthesized by PCR. Primer sequences are given in Additional file 1, Table S1. An amplified DNA fragment was α-^32^P-labeled using the Random Primers DNA Labeling System (Invitrogen). Membranes were prehybridized in ExpressHyb hybridization solution (Clontech, Mountain View, CA, USA) for two to four hours at 68°C, hybridized overnight at the same temperature in fresh ExpressHyb solution, washed according to the manufacturer's instructions and exposed to film.

### Fluorescence-activated cell sorting and analysis of cassette integration

FACS analysis was performed using BD FACSAria III cell sorter with BD FACSDiva software (BD Biosciences), and flow analysis was performed using a BD LSR II flow cytometer. Viable cells were gated on the basis of propidium iodide exclusion. At least 10,000 propidium iodide-negative events were analyzed. Untransfected cells were used as a control for baseline EGFP fluorescence.

### H3K9me3 profiling of endogenous retroviruses

To determine the H3K9me3 status of ERVs in TT2 wt versus *Setdb1 *KO mESC lines, we generated average H3K9me3 profiles for representative ERVs upregulated in the latter [19], including MusD, MMERVK10C, IAPEz, MLV and GLN elements. For each ERV family, all sequenced 50-bp reads from our previously published TT2 and *Setdb1 *KO H3K9me3 native ChIP-seq data sets [19] were aligned to the corresponding consensus sequences (including internal regions and corresponding LTRs) from Repbase http://www.girinst.org/repbase/[132] for all ERVs except MusD. For MusD, a representative active element was used (139824 to 132348 nt of AC084696, reverse strand). The Burrows-Wheeler Aligner http://bio-bwa.sourceforge.net/[133] was employed with default parameters (allowing up to two mismatches in the 32-bp seed and one gap). Reads were directionally extended by 150 bp, and extended reads were profiled along the element. All mapped reads were taken into account, and the profiles for each library were normalized by the total number of reads uniquely mapped to the *mm9 *genome. For reads that were aligned into multiple locations (LTRs), we considered only one randomly selected alignment location. The irregular nature of the profile is most likely attributable to SNPs and insertions and/or deletions in the consensus vs. genomic reads.

### H3K9me3 profiling in the sequences flanking endogenous retroviruses

To compare the average density of H3K9me3 in the genomic regions flanking ERVs, H3K9me3 N-ChIP-seq data sets for TT2 wt and *Setdb1 *KO mESCs [19] were used. Intact elements were selected for three ERV families: MusD, IAPEz, and MLV. For MusD, IAPEz and MLV, 195, 599 and 51 elements, respectively, satisfied the length and sequence similarity criteria that we applied [19]. All H3K9me3 reads that passed the quality score threshold above 7 were aligned to the mouse genome (*mm9*) using the Burrows-Wheeler Aligner [133] and directionally extended by 150 bp [19]. Only reads uniquely aligned to the regions within 7 kb on either side of intact elements were considered. If multiple reads were mapped to the same location, only one copy of the read was counted. To generate the profiles shown for the TT2 wt and *Setdb1 *KO cell lines, extended reads were first agglomerated for 5'- and 3'-flanks. Subsequently, the data were normalized to the total number of included elements and weighted by the total number of aligned reads to the genome for each sample.

## Abbreviations

BSA: bovine serum albumin; DMEM: Dulbecco's modified Eagle's medium; qPCR: quantitative polymerase chain reaction; RT: reverse transcriptase; siRNA: small interfering RNA; SNP: single-nucleotide polymorphism.

## Competing interests

The authors declare that they have no competing interests.

## Authors' contributions

IM carried out most of the research. PG performed ChIP-qPCR. JB and JPB derived the KO mESCs. MB performed bioinformatics analysis. DM and PS contributed reagents. IM and ML designed the study, analyzed the data and wrote the manuscript. All authors read and approved the final manuscript.

## Note added in proof

While this manuscript was under review, an article published by Shang and colleagues (PNAS 2011, 108(18):7541-7546) revealed that the H3K9me3 demethylase JMJD2B greatly facilitates H3K4 methylation by purified MLL2 *in vitro *(demonstrating that H3K9 demethylation is required for efficient H3K4 methylation) and is required for transcription of MLL2 targets *in vivo*.

## Supplementary Material

Additional file 1 **Figure S1**. Derivation of *Cbx5^-/- ^*mESCs via sequential targeted disruption of the *Cbx5 *gene. **Figure S2**. Derivation of *Cbx1^-/- ^*mESCs via sequential targeted disruption of the *Cbx1 *gene. **Figure S3**. Profiling of trimethylated lysine 9 of histone 3 (H3K9me3) along the length of endogenous retroviruses (ERVs). **Figure S4**. Profiling of H3K9me3 and H4K20me3 in the sequence flanking ERVs in wild-type and *Setdb1*-knockout mESCs. **Figure S5**. Knockdown (KD) of *Cdyl*, *Cdyl2*, *Chd4 *or *Mpp8 *does not result in reactivation of proviral reporters. **Figure S6**. Simultaneous KD of *Mpp8 *and *Cbx3 *does not result in reactivation of the ERV reporters. **Figure S7**. Proviral reporters are modestly reactivated upon KD of H3K9me3-binding H3K4 demethylases *Jarid1a-c*. **Figure S8**. Proviral reporters are modestly reactivated upon KD of H3K9me3-binding SRA (SET- and RING-associated) domain proteins *Uhrf1 *and *Uhrf2*. **Figure S9**. The level of derepression of the ERV reporters is substantially reduced in the *Setdb1*-KD cells following KD of the H3K4 methyltransferase *Wdr5*. **Table S1. Primers used in the study**. Click here for file
