# Supplementary material for: H3K9me3-binding proteins are dispensable for SETDB1/H3K9me3-dependent retroviral silencing
Source: Epigenetics Chromatin. 2011 Jul 20;4:12. doi: 10.1186/1756-8935-4-12 (PMC3169442; doi:10.1186/1756-8935-4-12)
Supplement: Additional file 1 — Figure S1. Derivation of Cbx5-/- mESCs via sequential targeted disruption of the Cbx5 gene. Figure S2. Derivation of Cbx1-/- mESCs via sequential targeted disruption of the Cbx1 gene. Figure S3. Profiling of trimethylated lysine 9 of histone 3 (H3K9me3) along the length of endogenous retroviruses (ERVs). Figure S4. Profiling of H3K9me3 and H4K20me3 in the sequence flanking ERVs in wild-type and Setdb1-knockout mESCs. Figure S5. Knockdown (KD) of Cdyl, Cdyl2, Chd4 or Mpp8 does not result in reactivation of proviral reporters. Figure S6. Simultaneous KD of Mpp8 and Cbx3 does not result in reactivation of the ERV reporters. Figure S7. Proviral reporters are modestly reactivated upon KD of H3K9me3-binding H3K4 demethylases Jarid1a-c. Figure S8. Proviral reporters are modestly reactivated upon KD of H3K9me3-binding SRA (SET- and RING-associated) domain proteins Uhrf1 and Uhrf2. Figure S9. The level of derepression of the ERV reporters is substantially reduced in the Setdb1-KD cells following KD of the H3K4 methyltransferase Wdr5. Table S1. Primers used in the study. [file 1756-8935-4-12-S1.PDF]

Supplementary Figure Legends

Figure S1. Derivation of *Cbx5*$^{-/-}$ ESCs *via* sequential targeted disruption of the *Cbx5* gene. To generate the *Cbx5*$^{-/-}$ mouse ESC line, we targeted each *Cbx5* allele (designated 1st and 2nd alleles) sequentially using a conditional vector ("HP1α cond. vector", left hand column) and a constitutive vector ("HP1α 1500 vector", right hand column), yielding "*Cbx5 gene (1st allele*)" and "*Cbx5 gene (2nd allele*)", respectively. Using primers a and c, a 2.6kb band was amplified by PCR from the wt allele. After targeting the *1st* allele, a diagnostic 4.4kb fragment (see agarose gel lane labeled "1st") was amplified, along with the wt allele. After electroporation with *Cre* mRNA, the *tk-neo*^{r} gene was excised from the HP1α cond. vector, yielding a reduced band size of 2.7kb (see agarose gel lane labeled "cre"), along with the wt band. Successful targeting of the *2nd* allele was validated using primers a and c, which yielded a diagnostic 3.7kb band (see agarose gel lane labeled "2nd"), along with the 2.7kb band from the 1st targeted allele after *Cre* deletion. As a final step, the targeted cells were electroporated with flpE mRNA to delete the 1st exon of HP1α in the "HP1α cond. vector" targeted allele. The absence of HP1α in the *Cbx5*$^{-/-}$ cells generated was confirmed by Western blotting (lane labeled "flpE"). Mock-electroporated cells (lane labeled "w/o flpE") were used as a positive loading control.

Figure S2. Derivation of *Cbx1*$^{-/-}$ ESCs *via* sequential targeted disruption of the *Cbx1* gene. (A) To generate the *Cbx1*$^{-/-}$ mouse ES cell line**,** we targeted each *Cbx1* allele (designated 1st and 2nd alleles) sequentially using targeting vectors with the same "arms" but possessing different selectable markers. The first *Cbx1* allele (see "*Cbx1* gene (1st allele)**")** was targeted using a vector constructed from the *Xho*I-*Hind*III genomic fragment of *Cbx1* into which a *tk-neo*^{r} gene was inserted (see "1st targeting vector"). Briefly, the unique *Sma*I site of exon 4 was engineered with a *Sma*I-*Not*I adapter that allowed the sub-cloning of the *tk-neo*^{r} gene on a *Not*I fragment. Exon 4 gives rise to bases 320 – 413 of the *Cbx1* mRNA (A of AUG given as 1) encoding amino acids 108 – 137 of HP1β, which lie adjacent to the C terminus of the CD [1]. Successful targeting was validated using a 0.3kb probe from the *Cbx1* gene (see grey box). After digestion of genomic DNA with *Bam*HI, this probe produced a fragment of 11.6 kb for the wild-type allele and 5.3 kb for the targeted allele (because of the introduction of a *Bam*HI site in the *Neo*^{r} gene; *C.f.* "*Cbx1* gene (1st allele)" with "Targeted 1st allele"). Once we had obtained a clone targeted

for the first allele, we then constructed a second targeting vector possessing the *pgk hyg*^{r} selectable marker (see "2nd targeting vector"). Briefly**,** the "1st targeting vector" was digested with *Not*I to release the *tk-neo*^{r} gene and the *pgk hyg*^{r} gene, to which *Not*I linkers had been added, was sub-cloned into the empty arms to give the 2nd targeting vector. The 2nd targeting vector was used to target the second *Cbx1* allele (see "*Cbx1* gene (2nd allele)") giving rise to the "Targeted 2nd allele", which yields a diagnostic 13kb band with *Bam*HI digestion. (**B**) Sequential targeting of the *Cbx1* gene was validated by Southern blotting. Genomic DNA from wt (left hand lane), *Cbx1*$^{+/-}$ (middle lane) and *Cbx1*$^{-/-}$ (right hand lane) ESCs was digested with *Bam*HI, run on a 1% agarose gel, blotted onto nitrocellulose and probed with a radio-labelled fragment of the *Cbx1* gene. Digestion of the wt allele yields a diagnostic 11.6kb band. Digestion of *Cbx1*$^{+/-}$ DNA yielded the wt band and a 5.3kb band indicative of the "Targeted 1st allele" shown in (A). Digestion of *Cbx1*$^{-/-}$ DNA resulted in both a 5.3kb band ("Targeted 1st allele") and a 13kb band, indicative of the "Targeted 2nd allele" shown in (A). **(C)** The absence of HP1β in the Cbx1-/- cells generated was confirmed by Western blotting, using protein extracts from wt, *Cbx1*$^{+/-}$ and *Cbx1*$^{-/-}$ ESCs. As expected, while *Cbx1*$^{+/-}$ cells express less HP1β than wt cells, *Cbx1*$^{-/-}$ cells show no detectable HP1β protein. No difference in HP1α protein levels are observed between wt, *Cbx1*$^{+/-}$, and *Cbx1*$^{-/-}$ cells. Western blotting and ECL detection was carried out as in [2].

Figure S3. H3K9me3 along the length of ERVs in wt and *Setdb1* KO mESCs. Profiles for MLV, MusD, MMERVK10C and GLN ERVs were generated by aligning all ChIP-seq reads from wt TT2 and *Setdb1* KO mESCs [3] to the consensus sequence of these ERVs, as described in the Materials and Methods section. H3K9me3 enrichment levels are presented as RPKM (reads per kilobase per million mapped reads).

Figure S4. H3K9me3 and H4K20me3 in the sequence flanking ERVs in wt and *Setdb1* KO mESCs. For profiling of H3K9me3 and H4K20me3 in the sequence flanking ERVs, we used H3K9me3 ChIP-seq reads from wt TT2 (C57BL/6 x CBA) and *Setdb1* KO mESCs [3] and H4K20me3 ChIP-seq reads from wt V6.5 (129SvJae x C57BL/6) mESCs [4], respectively. Reads were aligned to the genome (mm9), and the density of reads mapping to the genomic regions flanking intact MusD (159 elements) and MLV (51 elements) ERV families (7 kb 5' and 3' of the ERVs) was plotted for H4K20me3 (green) and H3K9me3 in wt (blue) and *Setdb1* KO mESCs (red). Vertical lines indicate the 5' and 3' boundaries of the ERV. The average

mappability for 50 bp reads was confirmed to be uniform in the flanks (data not shown), ruling out the possibility of mapping bias.

Figure S5. KD of *Cdyl, Cdyl2, Chd4 or Mpp8* does not result in reactivation of proviral reporters. To determine the percentage of cells with MSCV, MusD or IAP ERV reporter transgenes that are reactivated following KD of *Cdyl*, *Cdyl2*, *Chd4* and Mpp8, flow cytometry was carried out on day 5 post 2nd transfection with siRNAs specific for these genes as well as scrambled and *Setdb1*-specific siRNAs as negative and positive controls, respectively. GFP fluorescence data from at least 10,000 cells was collected for each sample.

Figure S6. Simultaneous KD of *Mpp8* and *Cbx3* does not result in reactivation of the ERV reporters. To determine the percentage of cells with MFG, MSCV, MusD or IAP ERV reporter transgenes that are reactivated following KD of *Mpp8 and Cbx3*, flow cytometry was carried out on day 5 post 2nd transfection with siRNAs specific for these genes, either alone or in combination, as well as scrambled and *Setdb1*-specific siRNAs as negative and positive controls, respectively. GFP fluorescence data from at least 10,000 cells was collected for each sample. Two independent biological replicates are shown.

Figure S7. Proviral reporters are modestly reactivated upon KD of H3K9me3-binding H3K4 demethylases *Jarid1A-C*. To determine the percentage of cells with MFG, MSCV, MusD or IAP ERV reporter transgenes that are reactivated following KD of *Jarid1A*, *Jarid1B* or *Jarid1C*, flow cytometry was carried out on day 5 post 2nd transfection with siRNAs specific for these genes, alone or in combination, as well as scrambled and *Setdb1*-specific siRNAs as negative and positive controls, respectively. Data from at least 10,000 cells was collected for each sample. Two independent biological replicates are shown.

Figure S8. Proviral reporters are modestly reactivated upon KD of H3K9me3-binding SRA domain proteins *Uhrf1* and *Uhrf2*. To determine the percentage of cells with MFG, MSCV, MusD or IAP ERV reporter transgenes that are reactivated following KD of *Uhrf1* and/or *Uhrf2*, flow cytometry was carried out on day 5 post 2nd transfection with siRNAs specific for these

genes, alone or in combination, as well as scrambled and *Setdb1*-specific siRNAs as negative and positive controls, respectively. Data from at least 10,000 cells was collected for each sample. Two independent biological replicates are shown.

Figure S9. The level of de-repression of the ERV reporters is substantially reduced in the *Setdb1* KD cells following KD of the H3K4 methyltransferase *Wdr5*. To determine the percentage of cells with MFG, MSCV, MusD or IAP ERV reporter transgenes that are reactivated following KD of *Wdr5* and *Setdb1 vs. Setdb1* alone, flow cytometry was carried out on day 5 post 2nd transfection with siRNAs specific for these genes, as well as scrambled siRNAs. Data from at least 10,000 cells was collected for each sample. Two independent biological replicates are shown.

References:

1. Ball LJ, Murzina NV, Broadhurst RW, Raine AR, Archer SJ, Stott FJ, Murzin AG, Singh PB, Domaille PJ, Laue ED: **Structure of the chromatin binding (chromo) domain from mouse modifier protein 1**. *The EMBO journal* 1997, **16**(9):2473-2481.

2. Aucott R, Bullwinkel J, Yu Y, Shi W, Billur M, Brown JP, Menzel U, Kioussis D, Wang G, Reisert I *et al*: **HP1-beta is required for development of the cerebral neocortex and neuromuscular junctions**. *The Journal of cell biology* 2008, **183**(4):597-606.

3. Karimi Mohammad M, Goyal P, Maksakova Irina A, Bilenky M, Leung D, Tang Jie X, Shinkai Y, Mager Dixie L, Jones S, Hirst M *et al*: **DNA Methylation and SETDB1/H3K9me3 Regulate Predominantly Distinct Sets of Genes, Retroelements, and Chimeric Transcripts in mESCs**. *Cell stem cell* 2011, **8**(6):676-687.

4. Mikkelsen TS, Ku M, Jaffe DB, Issac B, Lieberman E, Giannoukos G, Alvarez P, Brockman W, Kim T-K, Koche RP *et al*: **Genome-wide maps of chromatin state in pluripotent and lineage-committed cells**. *Nature* 2007, **448**(7153):553-560.

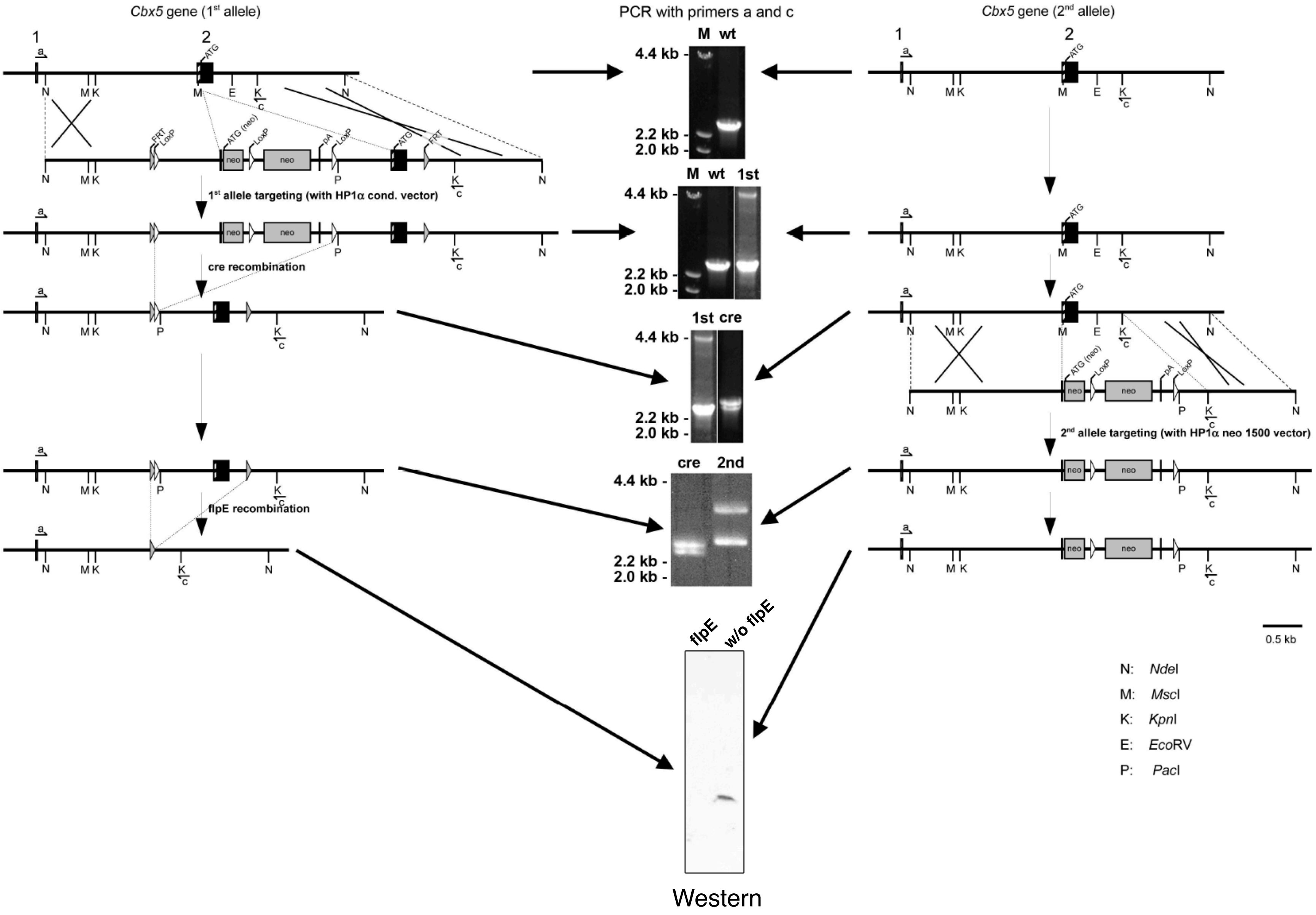

Maksakova et al. 2011

Supplementary Figure S1

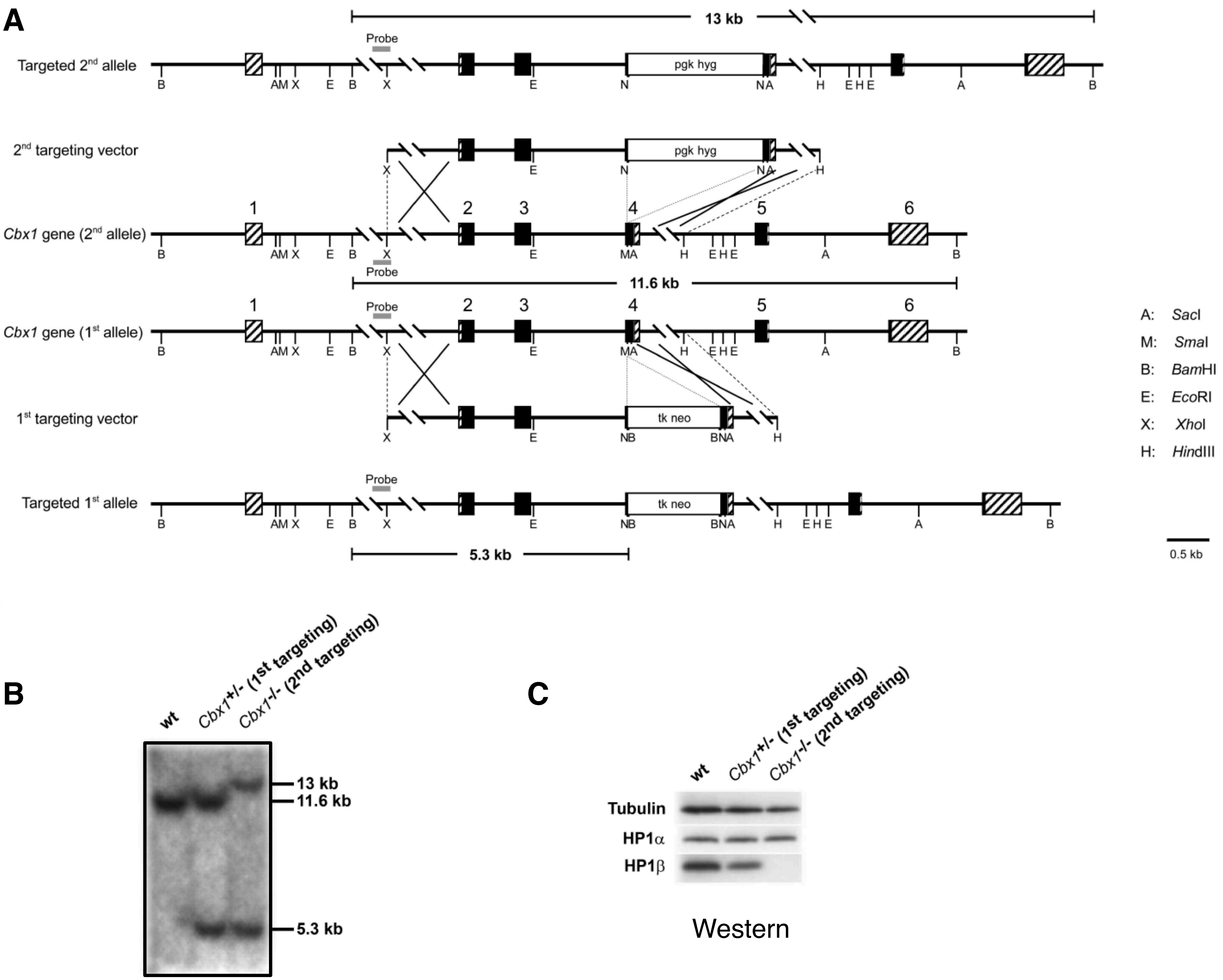

Maksakova et al. 2011

Supplementary Figure S2

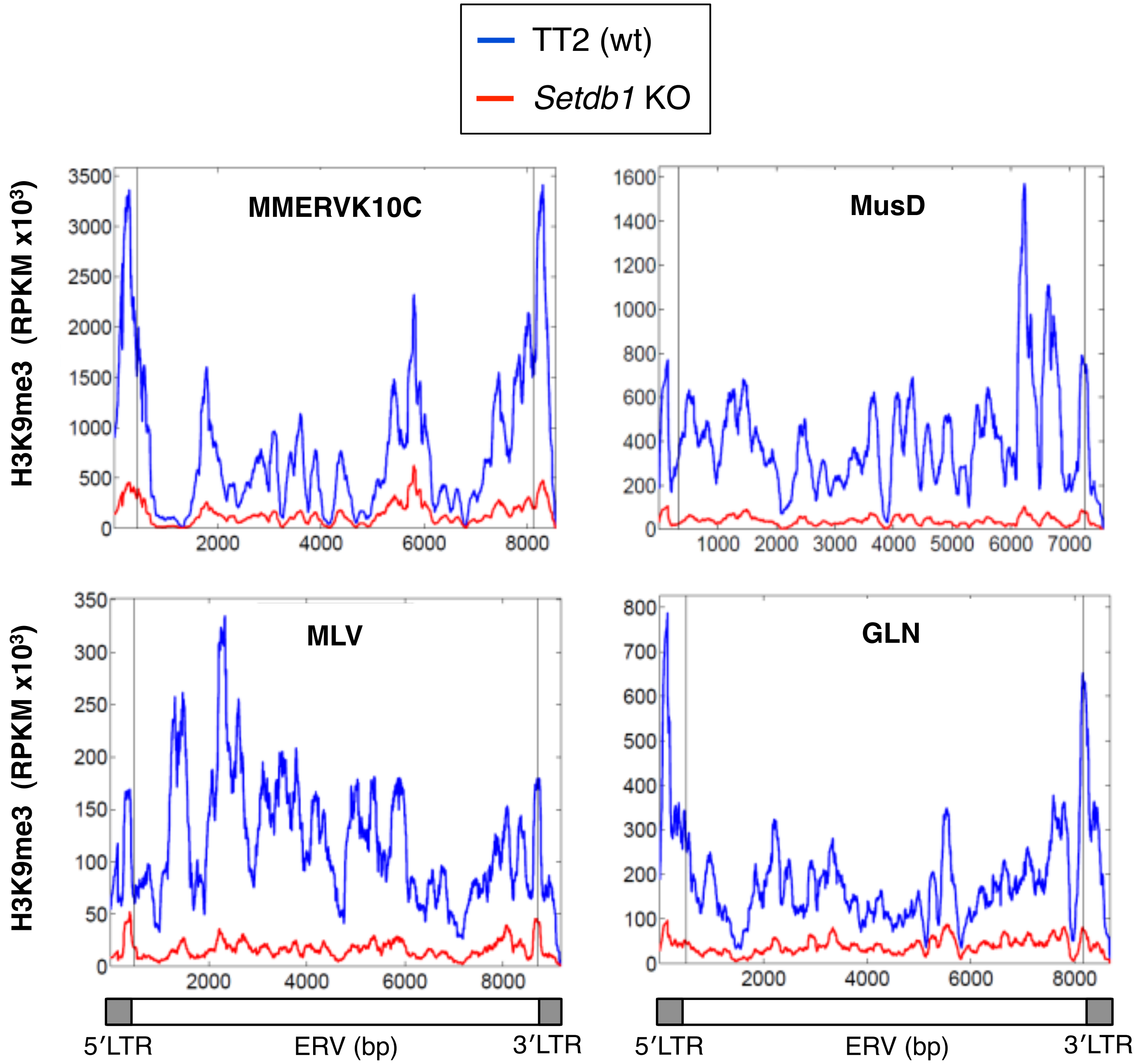

Maksakova et al, 2011

Supplementary Figure S3

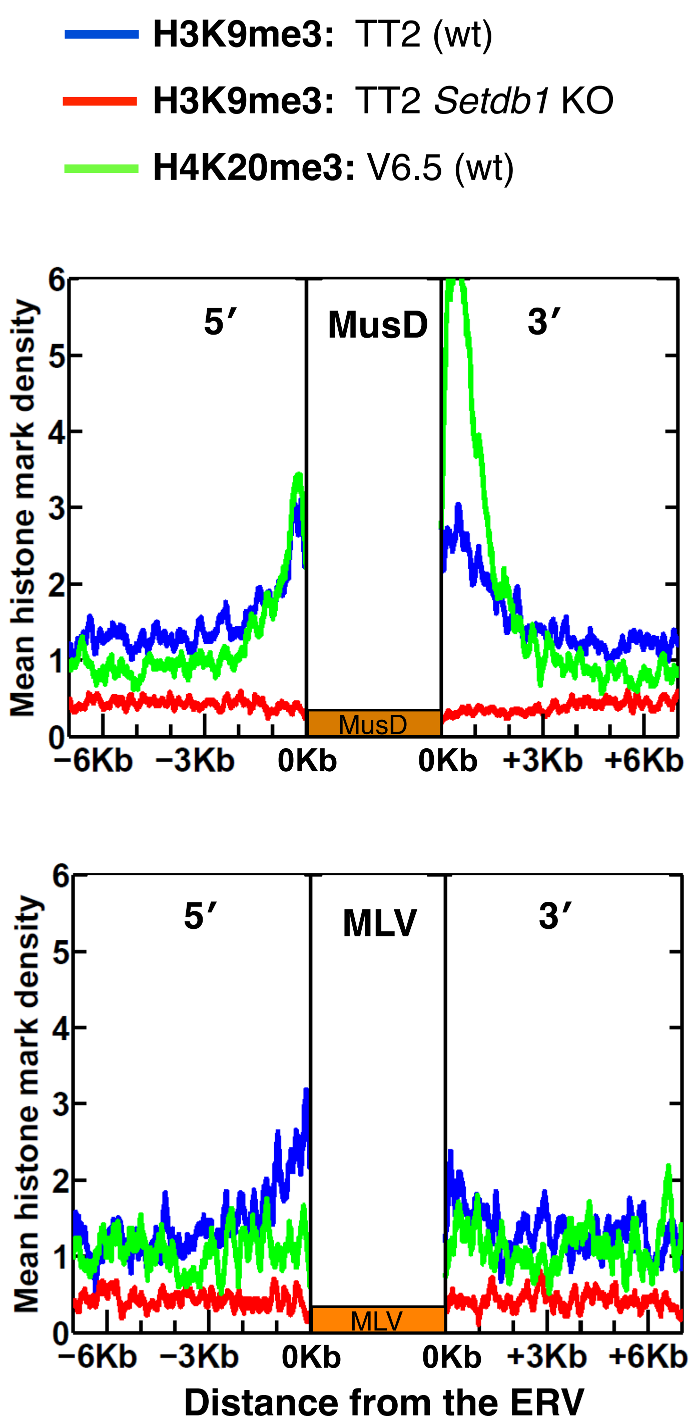

Maksakova et al, 2011

Supplementary Figure S4

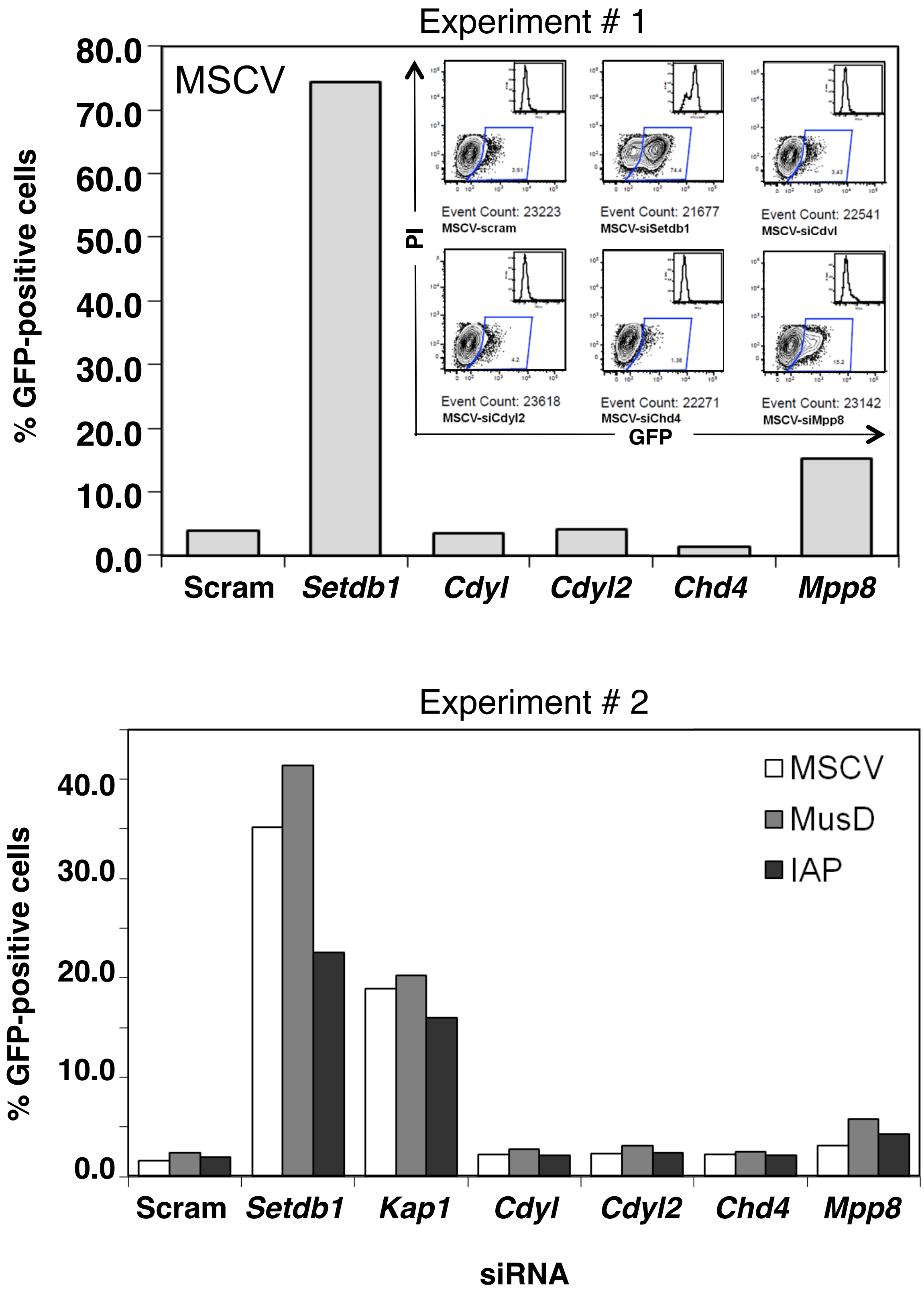

Maksakova et al, 2011

Supplementary Figure S5

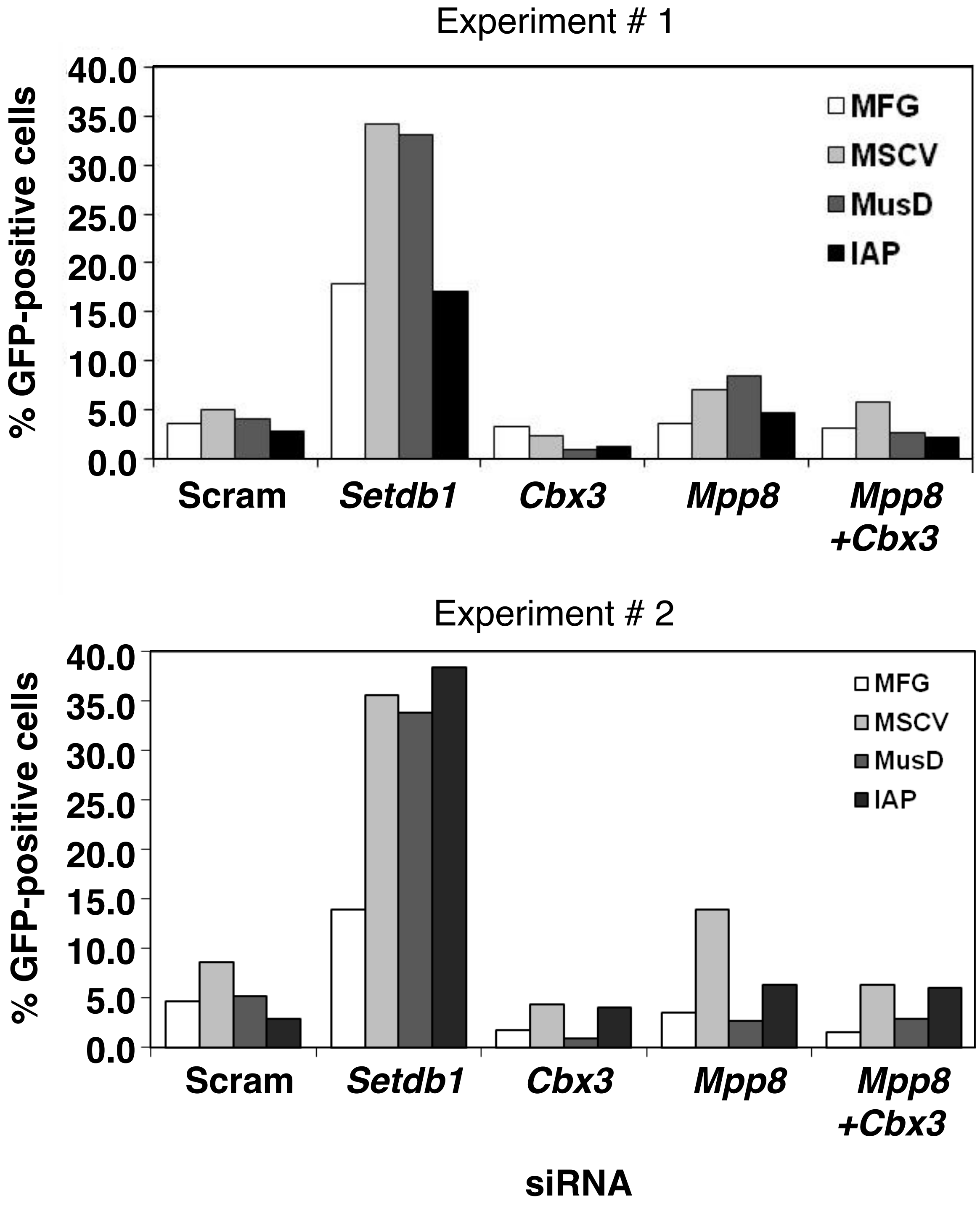

Maksakova et al, 2011 **Supplementary Figure S6**

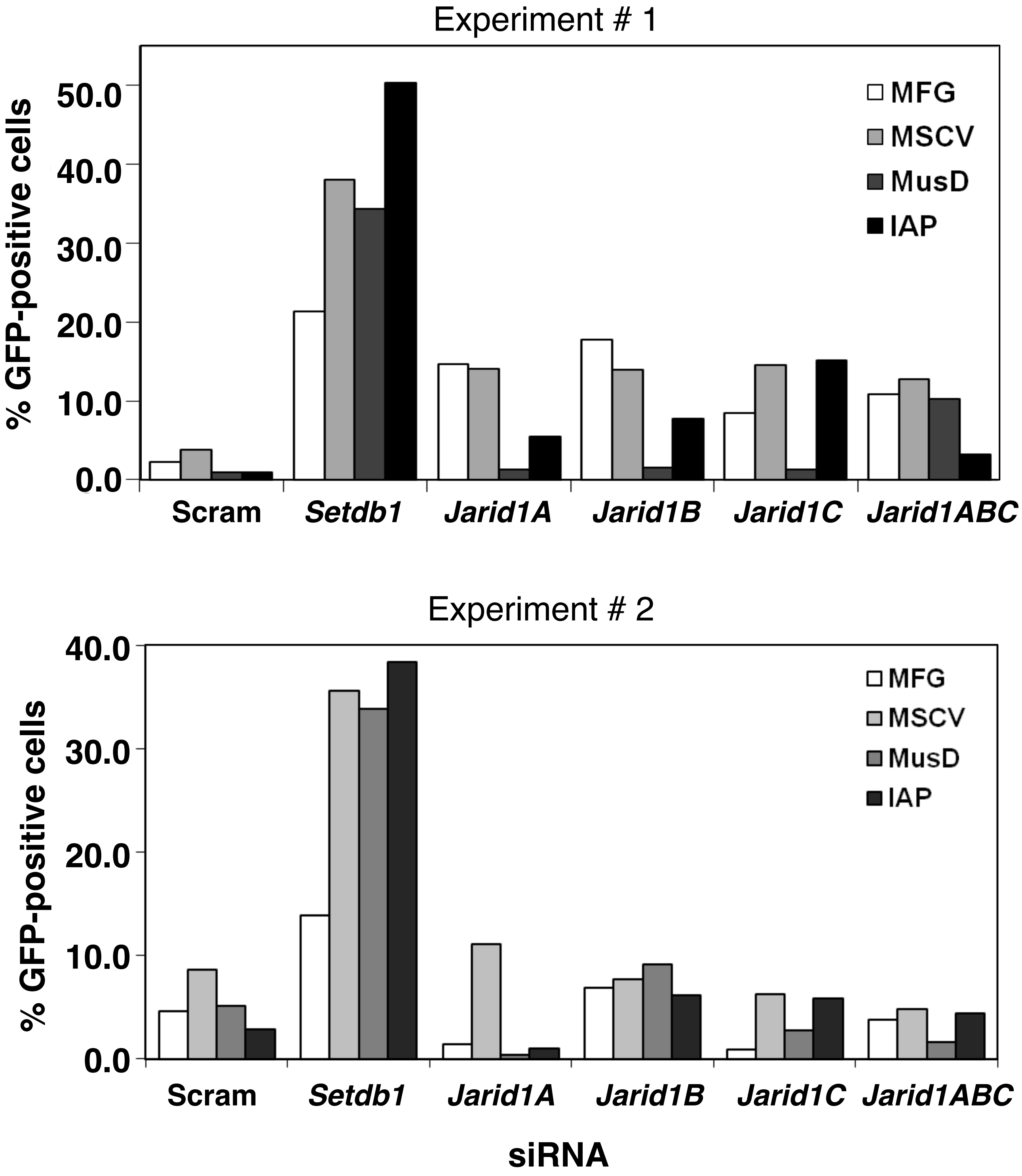

Maksakova et al, 2011 **Supplementary Figure S7**

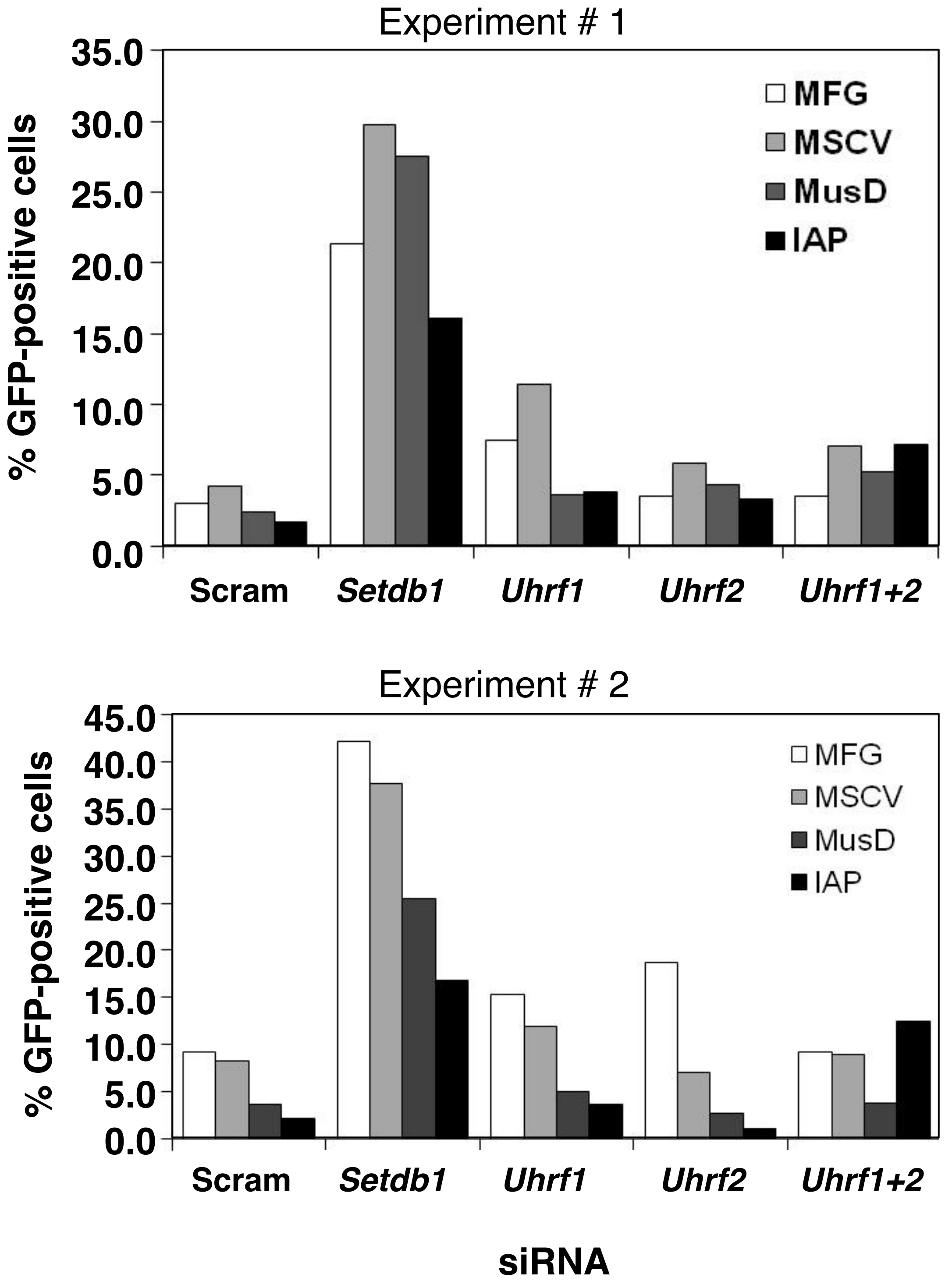

Maksakova et al, 2011 **Supplementary Figure S8**

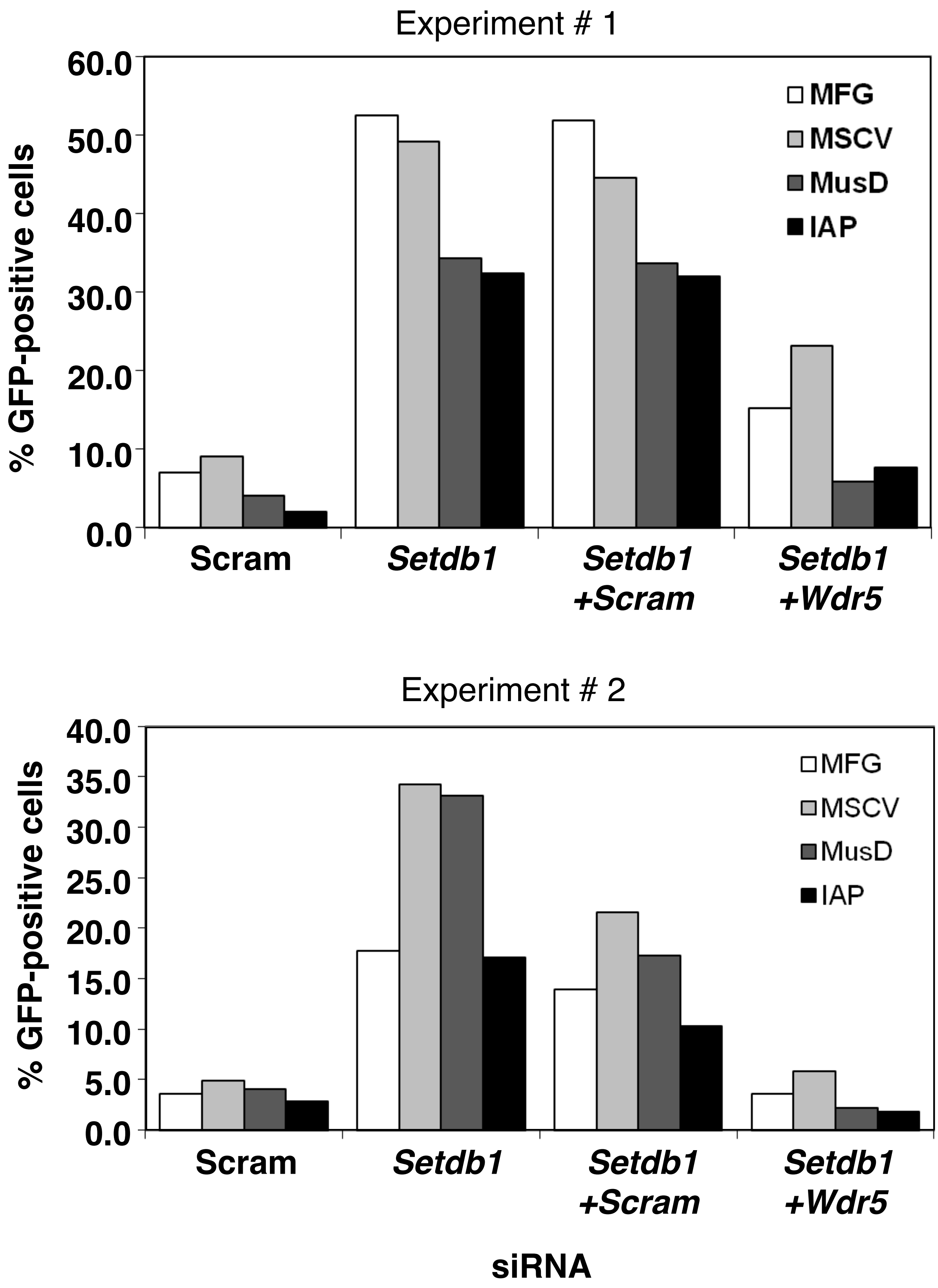

Maksakova et al, 2011

Supplementary Figure S9

Supplementary Table S1. Primers used in the study.

**Designation**	**Primer name**	**Primer sequence**
**RMCE cloning**		
MusD, 1st round	MusD_Chr8-131632864-292up	CATTGTGGGAATATCACCTTC
	MusD_768as-NheI	act*GCTAGC*-TTCCTTGAACCATGGGCAAC
MusD, 2nd round	MusD_1s-ClaI	*actATCGAT*-TGTAGTCTCCCCTCCC
	MusD_768as-NheI	act*GCTAGC*-TTCCTTGAA CCATGGGCAAC
IAP, 1st round	L62as	CCACACTAACTCTTAGCAGC
	IAP_803as-NheI	act*GCTAGC*-GACCCTTGGAAAGGCCTG
IAP, 2nd round	IAP_Wnt9b-1s-ClaI	*actATCGAT*-TGTGGGAAGCCGCCCC
	IAP_803as-NheI	act*GCTAGC*-GACCCTTGGAAAGGCCTG
**Bisulfite**		
ETnII/MusD	ETn/MusD_bis-40-s	GGGTGGAGTTTTTTGTTTATT
	ETn/MusD_bis-628-as	TTTCTTCTTCCACCTAAACAA
IAP	IAP2	GATAGTTGTGTTTTAAGTGGTAAATAAATA
	IAP4	ATTCTAATTCTAAAATAAAAAATCTTCCTTA
**N-ChIP qPCR**		
Maj Sat	MajF	GACGACTTGAAAAATGACGAAATC
	MajR	CATATTCCAGGTCCTTCAGTGTGC
MLV	End MLV-2	TGGGCAGGGGTCTCCAAATCT
	End MLV+2	ATAAAGCCTCTTGCTGTTTGCATC
IAP	IAP-LTR-ChIP-s	GCTCCTGAAGATGTAAGCAATAAAG
	IAP-retro-ChIP-as	CTTCCTTGCGCCAGTCCCGAG
MusD/ETnII	MusD&ETn2	CCCTTCCTTCATAACTGGTGTCGCA
	MusD&ETn3	TAGCATCTCTCTGCCATTCTTCAGG
IAP Chr2 #1	IAP Chr2 #1 F	AGAAGATTCTGGTCTGTGGTGT
	IAP Chr2 #1 R	TCTCAATTGGCTATAGTGTC
IAP Chr2 #2	IAP chr2 #2 F	ATGCCTGGAGACTGGCTAATG
	IAP chr2 #2 R	GTGTCAGGAAGAGACCACCA
IAP Chr2 #3	IAP chr2 #2.5 F	AGTTAGCTGAGACATCCTAACC
	IAP chr2 #2.5 R	TGAGGTTGAACCAGAATTTGA
IAP Chr2 #4	IAP chr2 #3 F	TCACCAGGTCCAGAGTCTAAC
	IAP chr2 #3 R	TCGCCAGGGCTCAGAGTCAC
IAP Chr5 #1	IAP chr5 #1 F	ATTCTGGTCTGTGGTGTTCTTCCT
	IAP chr5 #1 R	ATGGGCTAATGGCTTCCTGAGAT
IAP Chr5 #2	IAP chr5 #2 F	CTCATGAGCCACACCAGATCACT
	IAP chr5 #2 R	CTCACACAGACTCACAACAATG
IAP Chr5 #3	IAP Chr 5 #2.5F	TGGGAAAATGACAGAACTATT
	IAP Chr 5 #2.5R	GGAACCATCAAGAAACTCAC
IAP Chr5 #4	IAP chr5 #3 F	GTACACTCTTTCTCCTCAGACAT
	IAP chr5 #3 R	ATACTGAGTGTTGGGTCAGGCAAT
**qRT-PCR**		
MusD	ETn-MusD_514-s	GTGCTAACCCAACGCTGGTTC
	MusD_690-as	CTCTGGCCTGAAACAACTCCTG
MMERVK10C	MMERVK-10C_3854-fw	CAAATAGCCCTACCATATGTCAG
	MMERVK-10C_3985-rv	GTATACTTTCTTCTTCAGGTCCAC
IAPEz	IAP_4300s	AAGCAGCAATCACCCACTTTGG
	IAP_4400as	CAATCATTAGATG(T/C)GGCTGCCAAG

MLV	MLV2 fw	CAATAAAGCCTCTTGCTGTTTGCATC
	MLV2 rv	TGGGCAGGGGTCTCCAAATCT
GLN	GLN_631-fw	CGTAAGGACCCTAGTGGCTG
	GLN_784-rv	GCACTCACTCTTCTTCACTCTG
Setdb1	Setdb1-ex18-fw	CTTCTGGCTCTGACGGTGATG
	Setdb1-ex19-rv	GGAAGCCATGTTGGTTGATT
Cbx5	HP1a_ex2-fw	GGTTAAGGGGCAAGTGGAATATC
	HP1a_ex3-rv	CTCAGAAATTAGTTCAGGACAATCC
Cbx1	HP1b-ex3_s	AAGCGCAAAGCTGATTCTGATTC
	HP1b-ex4_as	TCAGTAGCTCCAATAATCCGCTC
Cbx3	HP1g_ex5-fw	ACAGACAGCAGCGGAGAGTTAA
	HP1g_ex6-rv	CTCGTAGAAGGCAATGACAATCTG
Cbx2	Cbx2_ex3-fw	AGAAGAGAACATTTTGGACCCGAG
	Cbx2_ex4-rv	AGGAGGATGTGACTGTGTGTTTC
Cbx7	Cbx7_ex4-fw	GAGCCAGAGGAGCACATCTTG
	Cbx7_ex2-rv	GTGGTTACCTGTAGCAGAAGCC
Cdyl2	Cdyl2_ex5-fw	CAGGTCTTCTGGCCCACGAC
	Cdyl2_ex6-rv	TCCAGCACTGACTTCAGGAAGC
Mpp8	Mpp8_ex2-fw	CATCTGGAGGACTGTAAGGAAGTTC
	Mpp8_ex3-rv	CTATCACTGTCTGCCTCAAATATGTC
Kap1	Kap1-5’	CGGAAATGTGAGCGTGTTCTC
	Kap1-3’	CGGTAGCCAGCTGATGCAA
Nanog	Nanog-qRT_ex3F	AAGTACCTCAGCCTCCAGCA
	Nanog-qRT_ex4R	CACCGCTTGCACTTCATCCT
β-actin	b-actin-ex2-fw	GAACCCTAAGGCCAACCGTG
	b-actin-ex3-rv	GGAGTCCATCACAATGCCTG
**Probes**		
ETnII/MusD	ETnII/MusD_465-s	GTAAAGTGTTGCTGAGGATG
	ETnII/MusD_652-as	ACCGCCTGATCCATGACTT
IAP	IAP_4300s	AAGCAGCAATCACCCACTTTGG
	IAP_4400as	CAATCATTAGATG(T/C)GGCTGCCAAG
Gapdh	mGAPDH_139s	AACGACCCCTTCATTGAC
	mGAPDH_331as	CTCCACGACATACTCAGCAC
